# Pathways to strengthen the climate resilience of health systems in the Peruvian Amazon by working with Indigenous leaders, communities and health officers

**DOI:** 10.1136/bmjgh-2023-014391

**Published:** 2024-09-07

**Authors:** Claudia L Vidal-Cuellar, Victoria Chicmana-Zapata, Ingrid Arotoma-Rojas, Graciela Meza, James D Ford, Hugo Rodríguez Ferruchi, Elida De-La-Cruz, Guillermo Lancha-Rucoba, Diego B Borjas-Cavero, Sonia Loarte, Ofelia Alencastre Mamani, Victoria I Peña Palma, Maria G Coronel-Altamirano, Ivonne Benites, Giovanna Pinasco, Rosa Valera, Marco Maguiña Huaman, Adolfo Urteaga-Villanueva, César V Munayco, Carol Zavaleta-Cortijo

**Affiliations:** 1 Unidad de Ciudadanía Intercultural y Salud Indígena (UCISI), Facultad de Salud Pública y Administración, Universidad Peruana Cayetano Heredia, San Martín de Porres, Peru; 2 The Priestley Center for Climate Futures, University of Leeds, Leeds, UK; 3 Faculty of Medicine, Universidad Nacional de la Amazonia Peruana, Iquitos, Peru; 4 Organización de Mujeres Indígenas Amazónicas Asháninkas de la Selva Central (OMIAASEC), Satipo, Peru; 5 Unidad de Ciudadanía Intercultural y Salud Indígena (UCISI), Facultad de Salud Pública y Administración, Universidad Peruana Cayetano Heredia, Yurimaguas, Peru; 6 Dirección de Promoción de la Salud, Estado Peruano Ministerio de Salud, Lima, Peru; 7 Dirección de Pueblos Indígenas u Originarios, Estado Peruano Ministerio de Salud, Lima, Peru; 8 Dirección de Salud Mental, Estado Peruano Ministerio de Salud, Lima, Peru; 9 Dirección de Gestión de Riesgos y Desastres (DIGERD), Estado Peruano Ministerio de Salud, Lima, Peru; 10 Coordinadora Regional Salud de Pueblos indígenas, Gerencia Regional de Salud Loreto, Iquitos, Peru; 11 Estrategia de Pueblos Indígenas u Originarios, Red de Salud de Satipo, Satipo, Peru; 12 Oficina de Emergencias y Desastres, Red de Salud de Satipo, Satipo, Peru; 13 Centro Nacional de Epidemiología, Prevención y Control de Enfermedades (CDC), Estado Peruano Ministerio de Salud, Lima, Peru

**Keywords:** Health systems, Environmental health, Health services research, Health policies and all other topics

## Abstract

**Background:**

Indigenous knowledge and responses were implemented during the COVID-19 pandemic to protect health, showcasing how Indigenous communities participation in health systems could be a pathway to increase resilience to emergent hazards like climate change. This study aimed to inform efforts to enhance climate change resilience in a health context by: (1) examining if and how adaptation to climate change is taking place within health systems in the Peruvian Amazon, (2) understanding how Indigenous communities and leaders’ responses to climatic hazards are being articulated within the official health system and (3) to provide recommendations to increase the climate change resilience of Amazon health systems.

**Methods:**

This study was conducted among two Peruvian Amazon healthcare networks in Junin and Loreto regions. A mixed methodology design was performed using a cross-sectional survey (13 healthcare facilities), semistructured interviews (27 official health system participants and 17 Indigenous participants) and two in-person workshops to validate and select key priorities (32 participants). We used a climate-resilient health system framework linked to the WHO health systems building blocks.

**Results:**

Indigenous and official health systems in the Peruvian Amazon are adapting to climate change. Indigenous responses included the use of Indigenous knowledge on weather variability, vegetal medicine to manage health risks and networks to share food and resources. Official health responses included strategies for climate change and response platforms that acted mainly after the occurrence of climate hazards. Key pathways to articulate Indigenous and official health systems encompass incorporating Indigenous representations in climate and health governance, training the health work force, improving service delivery and access, strengthening the evidence to support Indigenous responses and increasing the budget for climate emergency responses.

**Conclusions:**

Key resilience pathways call for a broader paradigm shift in health systems that recognises Indigenous resilience as valuable for health adaptation, moves towards a more participatory health system and broadens the vision of health as a dimension inherently tied to the environment.

**WHAT IS ALREADY KNOWN ON THIS TOPIC**

- Climate change is influencing the intensity and frequency of extreme climate events in the Amazon region.
- Health risks associated with climatic hazards may be preventable by building climate-resilient health systems.
- Indigenous peoples have demonstrated resilience in facing multiple socioenvironmental shocks over millennia.

**WHAT THIS STUDY ADDS**

- Indigenous responses inform climate change health system resilience, especially adding practices for the transformation of the health system.
- This study found that multiple Indigenous responses were not yet articulated within the official health systems.

**HOW THIS STUDY MIGHT AFFECT RESEARCH, PRACTICE OR POLICY**

- A transformation of the current health systems paradigm is needed to work in multicultural settings by recognising, using and valuing Indigenous knowledge to inform local health systems and promote sustainable responses to climate change impacts.

## Introduction

 Climate change is a major threat to human health, and health systems need to be prepared to respond to the direct and indirect impacts of changing climatic conditions.^1^ Peru is a country highly vulnerable to climate change because of its geography, which includes more than 70% of the types of climates worldwide, an economy that depends on the production and use of fossil fuels, and a population with more than 50 different Indigenous peoples, who rely on natural ecosystems for their livelihoods, culture and well-being.^2^ In the Amazon region, climate change is acting as an emergent environmental stressor, projected to contribute to significant ecological transformations,^3^ and is influencing the intensity and frequency of extreme climate events.^4^

For Peruvian Indigenous Amazonian populations—like for many Indigenous peoples globally—the concept of ‘health’ extends beyond individual physical well-being and focuses strongly on harmonious relationships with nature and community.^5^,^7^ Previous research shows that Indigenous peoples’ holistic vision of health has enabled them to overcome challenges and create solutions to protect health within their communities during global emergencies. For instance, during the COVID-19 pandemic, Indigenous peoples responded by using and protecting natural resources, identifying medicinal plants and reviving knowledge to protect food security.^8^ Despite efforts by the official health system to offer culturally appropriate healthcare for Indigenous communities through intercultural health policies and community participation strategies, the provision of services still faces serious limitations. The low coverage of healthcare services, poor infrastructure, shortages of material and human resources and a lack of cultural sensitivity training stand out as important challenges to delivering healthcare services in Indigenous communities.^9^,^11^ Similarly, sub-national governments often face challenges in effectively supporting health systems and climate change adaptation. These difficulties include inadequate collaboration with both government and non-government actors,^12^ political instability that performs as a driver to increase the risk of climate hazards,^13^ and climate change mitigation policies (eg, conservation) that jeopardise Indigenous rights and land ownership.^14^

In this context, community engagement approaches informed by Indigenous knowledge systems have been recommended as a measure to accelerate climate change adaptation^15 16^; however, there is limited evidence on how to articulate Indigenous responses within formal governmental responses to increase the climate resilience of health systems.^17^ Peru’s National Adaptation Plan (NAP)—formulated to identify adaptation needs and develop and implement strategies and programmes to address those needs—prioritises health as one of the five sectors of mainstream climate adaptation and recognises that Indigenous populations are highly vulnerable to climate change.^2^ Although Peru’s NAP proposes an intercultural approach to be used across the five sectors, there is scarce information on how the Indigenous community’s responses can inform official health systems in a climate change context. A better understanding of this articulation can increase the climate resilience of the health systems in Peru and other countries with multiple Indigenous peoples living in Amazonian regions.

This study aimed to inform the climate change resilience of health systems in the Peruvian Amazon by working with Indigenous leaders, community members and health officers from two Amazon regions: Loreto and Junin. Our specific objectives were: (1) to understand whether the regional health system is adapting to climate change, (2) to understand how Indigenous communities and leaders’ responses to climate shocks are being articulated with the official health system and (3) to provide recommendations to increase the climate change resilience of Amazon health systems.

## Methods

### Theoretical framework

We used a resilience framework based on climate change resilience and health systems building blocks, informed by the WHO. The resilience approach allowed us to identify vulnerabilities and specific responses to environmental risk, and to understand the process of addressing them to build the capacity to manage change.^15 18 19^ A resilience approach aligns with systems thinking that recognises the complexity of health, its risk factors and the existence of multiple sub-systems; embraces non-linearity to identify synergies and amplifiers; and anticipates and mitigates potential negative consequences on human health.^20^ We used the Standards for Quality Improvement Reporting Excellence checklist when writing our report.^21^

#### Climate change health resilience

This study used a definition of climate-resilient health systems proposed by WHO: ‘A climate resilient health system is one that is capable of anticipating, responding to, coping with, recovering from and adapting to climate-related shocks and stress, so as to bring sustained improvements in population health, despite an unstable climate’.^22^ Along the study, five dimensions were investigated: (1) climate hazards or shocks to health, (2) individuals, communities and systems exposed, (3) responses and adaptations taken, (4) vulnerability to climate hazards of human health systems, considering multiple socioeconomic and environmental amplifiers and/or modulators of climate hazards impacts on health systems and (5) health risks and exposure pathways. These dimensions were aligned with the recommendations of the Intergovernmental Panel on Climate Change about health risks to climate change^23^ and the WHO climate change and health vulnerability and adaptation assessment.^24^

#### Health systems resilience building blocks

The climate health resilience approach was applied in connection with the ‘building blocks’ that WHO had proposed for building climate-resilient health systems.^20 22 25^ We focused the analysis on how different components of the Amazon regional health system engaged with Indigenous and non-Indigenous responses to adapt and to build resilience.

### Study setting

#### Geographical and climatic characteristics

The Peruvian Amazon comprises 60% of Peru’s territory^26^ and is characterised by a tropical climate with high temperatures and frequent precipitation throughout the year.^27^ The weather typically features two seasons: a wet season (January–March), with particularly intense rainfalls, and a dry season (July–August), with lighter rainfalls.^28 29^ In the last decades, variations in the duration and intensity of these seasons have been documented and consistently attributed to climate change.^26^ From the 1960s up to the end of the last century, precipitation in the Peruvian Amazon decreased by 20%–30%, while maximum and minimum temperatures increased by 0.2°C/decade. Future projections for the Peruvian Amazon indicate a decrease in precipitations of 10% and a rise in maximum temperatures of 1.6°C (0.53°C/decade) by 2030 and of 3°C by 2050.^30 31^

**Figure 1:**
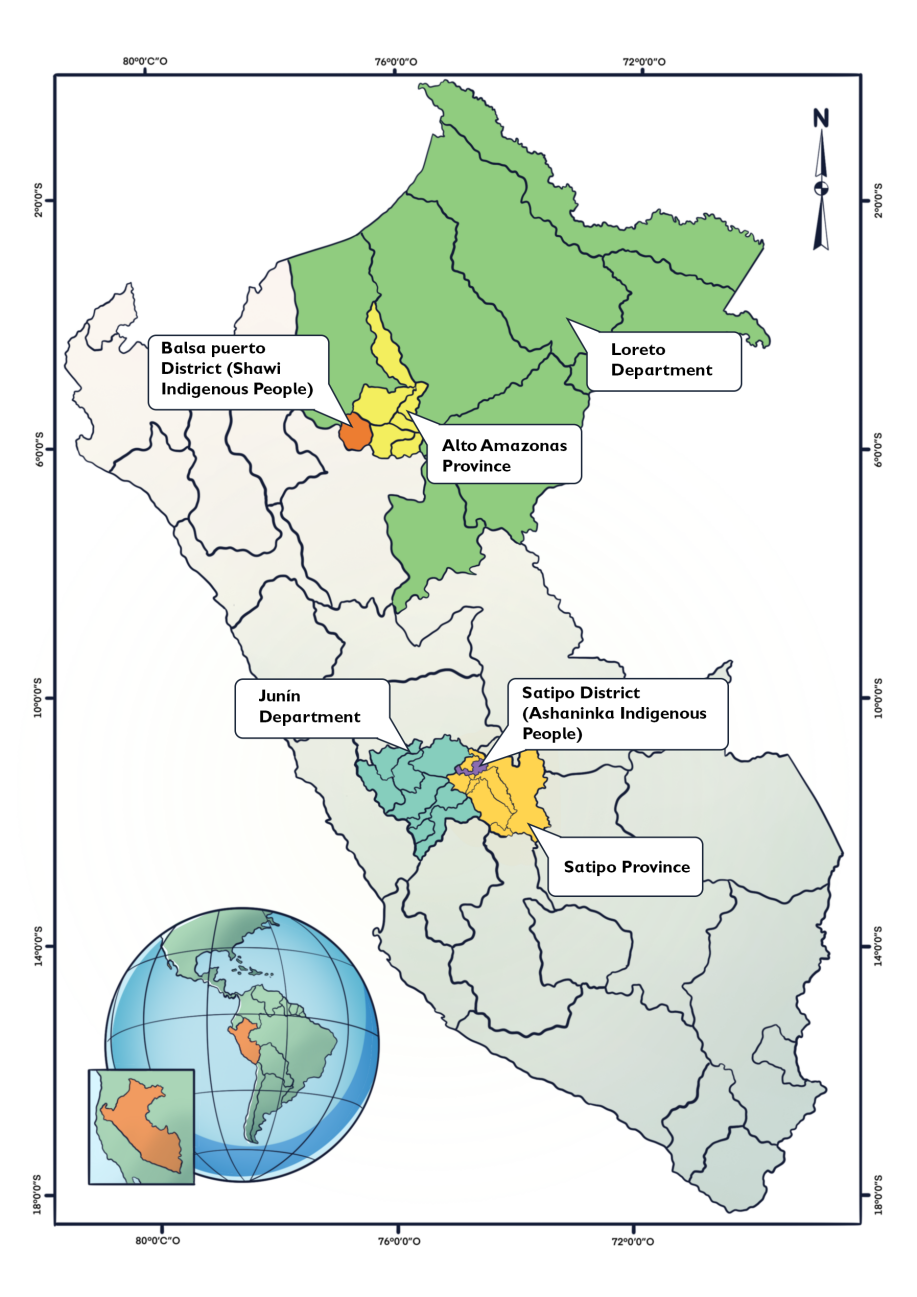
Map to show the two locations where this study was conducted in the Peruvian Amazon.

Our study was set in two regions of the Peruvian Amazon: Loreto and Junin. Both regions were considered at high risk in the face of climate change according to the Peruvian national health risk climate change assessment.^27^ Besides, these regions were selected for our study on consultation with our Indigenous collaborators and build on longstanding partnerships between the research team and Indigenous communities of these zones (figure 1).

#### Official health system: health networks

In Peru, each Amazon region structures its healthcare system on multiple health networks. Peruvian health networks are a health system model that aims to deliver equitable access and integral healthcare services to a population localised in a particular geographical area.^32 33^ This study was focused on two Amazon health networks: Alto Amazonas (Balsapuerto district, in the Loreto region) and Satipo (Satipo district, in the Junin region), and within those health networks we worked with two Indigenous populations: the Shawi in Balsapuerto and the Ashaninka in Satipo district.

Amazon health networks mainly contain two types of primary care facilities: health posts and health centres. Health posts are the first point of access to healthcare for rural populations, located in towns with <1000 inhabitants, with poor communication infrastructure and difficult geographical access.^34^ Health centres are located in a provincial or district capital, under the direction of a physician, and equipped to make diagnostic tests. In both the Loreto and Junin regions, primary care institutions represent more than 97% of healthcare facilities.^35 36^ In the Balsapuerto district, the Alto Amazonas health network has 17 healthcare facilities (14 of which are health posts), a population of 15 723 inhabitants. In the Satipo district, the Satipo health network includes 9 healthcare facilities (8 of which are health posts), and provides attention to 29 Indigenous communities.

#### Indigenous health system

In parallel to the official health system, in the Peruvian Amazon, there is a non-official sub-system formed by Indigenous peoples who provide care for community members, which is relevant for its cultural importance and practical implications for bringing preventive and recuperation health support in remote settings. On one hand, Community Health Agents (CHAs)^37^ are volunteer community members native to local communities who have received basic training in health promotion from the Ministry of Health or local non-governmental organisations (NGOs). CHAs represent a nexus between the official health system and the Indigenous populations. On the other hand, there are non-official health providers who are part of the Peruvian Amazon ancestry medical system, which is still prominent in Peru, they are called in Spanish ‘vegetalistas’, ‘curanderos’, ‘shamans’, among other terms.^5^ A recent study found that more than half of the people visiting an Amazon health facility went to seek health attention from a Shaman or used medicinal plants to solve their health conditions first.^38 39^

### Design

We used a mixed methodology convergent design comprising three types of methods: a cross-sectional survey, semistructured interviews and in-person workshops. Information from the different methods allowed for the triangulation of results^40^ and was presented through a narrative description. Our mixed-method approach was built on quantitative and qualitative components. The quantitative component was the cross-sectional survey, and the qualitative study relied on Grounded Theory^41^ supported by semistructured interviews and in-person workshops. Grounded Theory seeks to investigate social interactions and experiences to explain a process, by using a flexible, iterative, inductive and comparative analysis.^42^

The Grounded Theory helped to inform the WHO framework on climate change and health systems resilience by providing information about people who are on the ground, in the case of this study, Indigenous and non-Indigenous participants from Peruvian Amazon regions. The multiple methods that we have used allow us to revise our findings and validate them with the participants. For example, in the interviews, we identified emerging themes by the follow-up questions that were asked to the participants to complement and explain the topics based on their own perceptions. Also, during the dissemination workshop, participants identified priorities and provided feedback that was then translated into the results of the study. This design enabled the iterative revision of the emerging themes and helped us to identify recommendations to inform the WHO framework with the lived experiences of people directly impacted by climate change—these matters are reported in the Discussion section.

### Participants

#### Official health system

Participants of the official health system were defined as people who work at organisations related to health and/or other institutions in Loreto or Junin regions, involved in sectors like agriculture, disaster offices, culture, municipalities and others, according to the context of each region. We included participants with at least 2 years of experience working in their institution to ensure they are aware of how the local health systems work.

#### Indigenous health system

Participants of the Indigenous health system were defined as people who self-recognise as Indigenous (Shawi or Ashaninka), 18 years or older, who live in an Indigenous community and who can provide information relevant to the topic of interest, regardless of their sex, educational achievement or other titles that do not necessarily apply to the Indigenous way of life. They could be Indigenous leaders, community health agents, Indigenous healers or Indigenous midwives.

We worked with collaborators of the following Indigenous organisations: Organizacion de Mujeres Indigenas Amazonicas de la selva Central (OMIAASEC), in Junin and Gobierno Territorial Autónomo de la Nación Shawi, in Loreto. Building on the trust of our previous work,^8 43^ our Indigenous partners helped and joined us to complete the visits to the communities. Only when we had the interest and permission of the potential Indigenous participants, a formal letter from Universidad Peruana Cayetano Heredia was prepared to invite them to be interviewed.

### Instruments

#### Semistructured interviews

##### Sampling

To select participants for our interviews, we applied actor mapping based on Waweru *et al* to identify key actors to be interviewed. The actor mapping was applied to the official in charge of the Indigenous offices in Loreto and Satipo health networks. Finally, the semistructured interviews were applied in person to members of both health systems: the Indigenous (n=17) and the official health system (n=27) in Loreto and Junín.

##### Instruments, data collection and analysis

Our semistructured interviews were aimed to characterise the capacity of local health systems to respond to key climate health hazards and on community engagement. Our instrument explored lived experiences with an extreme climate event(s) in the past, and how the health systems responded to handle it. For cases where participants were not able to remember a past extreme climatic event, we used a recent extreme health hazard like the COVID-19 pandemic, to understand responses and community engagement. One participant out of 17 Indigenous participants was not able to remember a past extreme climatic event and talked about COVID-19. Online supplemental material 1 shows qualitative instrument.

Data collection was performed by a sociologist and a physician, with an Indigenous research assistant of the ethnicity that is predominant in the region, Shawi or Ashaninka. When participants preferred to speak in the Indigenous language, translations to Spanish were conducted in the presence of each participant to corroborate answers. Data from the interviews were analysed with NVivo V.12 software. Thematic analyses included the creation of descriptive and analytic codes based on the dimensions in the theoretical framework. Online supplemental material 2 shows the lists of codes created a priory and after revising the interviews. The iterative process of data revision allowed for identifying new emergent sub-themes related to ‘meanings’, ‘process’ and ‘definitions’ handled by participants especially to understand how to favour the articulation of Indigenous responses to shocks with the official health systems, and to describe the climate change adaptations that health systems are being taken. For example, the three dimensions of vulnerability (geographical, socioeconomic and environmental) to climate hazards emerged after the revision of data. Also the process of ‘building resilience’ as a combination of multiple mechanisms of responses emerged after revision and triangulation.

### Cross-sectional survey

#### Sampling

A cross-sectional survey was applied to healthcare facilities of the health networks Alto Amazonas (n=6) and Satipo (n=7). Purposeful sampling was used with a remoteness criterion, using the distance from the main urban centre where the head of the health network is located. These criteria were validated by the Indigenous officers in the health networks of both regions to ensure an acceptable diversity of healthcare facilities.

#### Instruments, data processing and analysis

We designed the survey to characterise the health systems on the ground and investigate how the process of articulation between Indigenous and non-Indigenous health representatives was conducted. Our instrument enquired about the basic infrastructure of the health facility, service delivery, health priorities and community engagement, see online supplemental material 3. Members of our research team applied the survey to the responsible/head of the health facility by using Kobo Toolbox (kf.kobotoolbox.com) from smartphones. The survey could be easily accessed without an internet connection, and the average time to complete it was approximately 15 min. Collected data were transferred from the Kobo toolbox to Microsoft Excel 2019 to check for data completeness and perform data cleaning. Thereafter, we exported the data to STATA software V.17.0 for statistical analysis. We performed a descriptive analysis by calculating frequencies and percentages only, considering that our study population size was small and our aim was to complement data obtained from other methods.

### In-person workshops

We performed one workshop in each region to bring together the members of the Indigenous and official health systems who participated in our study. The objectives of the workshop were to present the preliminary results of our study and validate them; identified challenges for climate adaptation and discussed factors that could make the regional health networks more prepared to manage climatic effects on health by taking into account Indigenous people’s responses. At the end of the workshop, three work priorities to increase the resilience of the health systems of each region were identified. A total of 32 participants attended to the workshops. A breakdown of the participants in the workshops can be found in online supplemental material 4.

**Figure 2:**
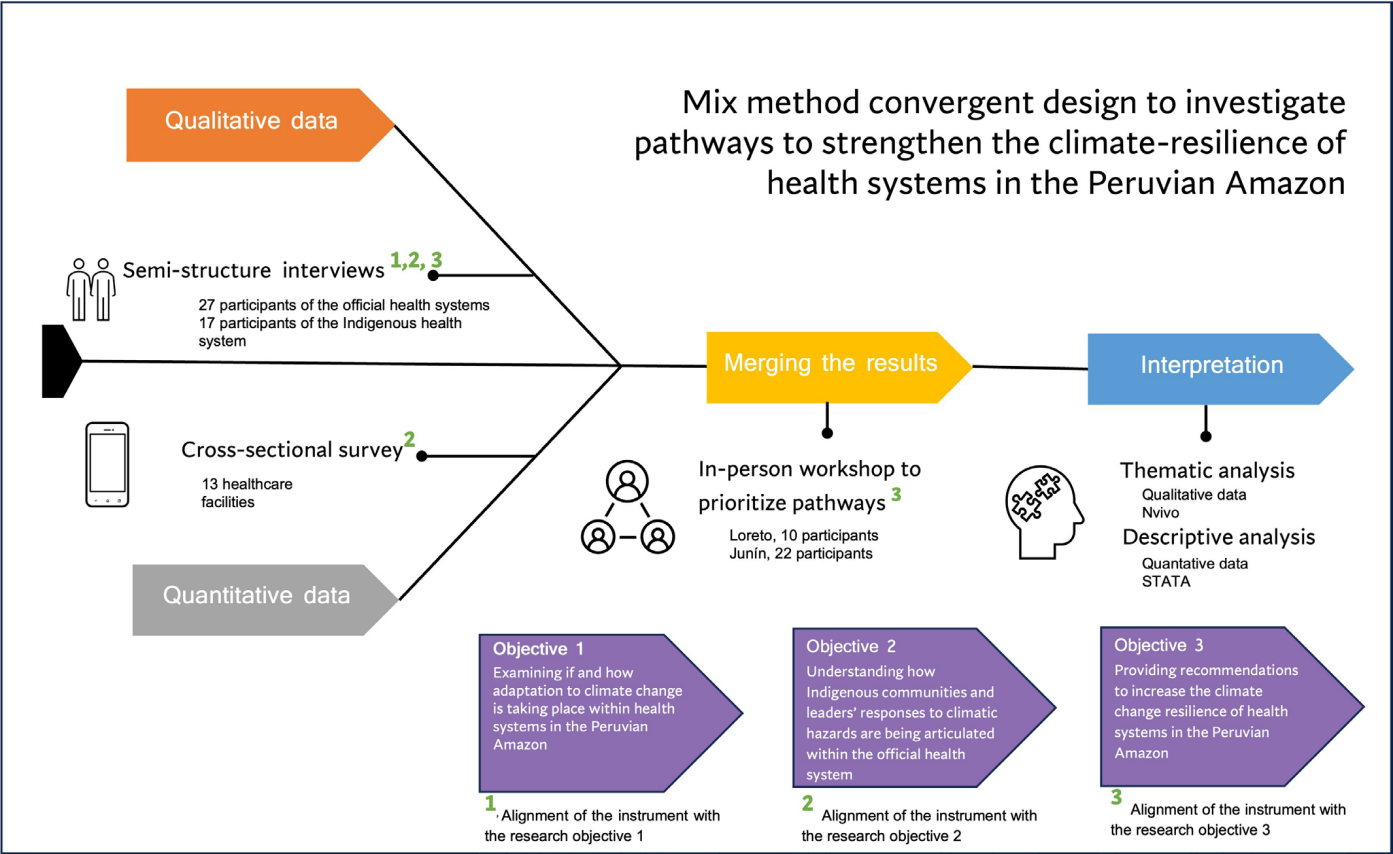
Mixed methodology convergent design with specific instruments and research objectives.

A summary of how each instrument used in this study was aligned with our mixed methodology convergent design is shown in figure 2.

### Ethics

A consent form was prepared to discuss the extent of the interview and the purpose of the study.

We maintained regular contact with Indigenous organisations by sharing with them monthly reports about the research progress and updates. Also, we coordinated with our Indigenous organisation partners in each region to develop a workshop on climate change and health at the end of the study as part of a collective benefit for the participants of the Indigenous health system. For our participants in the official health system, we delivered a short course on climate change and health.

### Patient and public involvement

This study was conceived during our previous work with Indigenous leaders on the topic of responses to COVID-19. Indigenous leaders contributed by suggesting names of Indigenous community members to be appointed as Indigenous researchers in their regions. During the development of the research protocol, we submitted the study to the Peruvian Ministry of Health inviting them to provide insights and comments. As a result, a workgroup was formed with the Directorate of Disaster Risk Management of the Ministry of Health of Peru to generate regular project updates, receive inputs and present the results monthly. The workgroup was composed of national health officers from the Ministry of Health (including Climate Change, Health promotion and Indigenous peoples offices) and local health officers from the regions of study. In addition to the research process, the workgroup supported us in completing the delivery of a course on climate change and health to benefit the participants of the official health system. Indigenous research assistants and Ministry of Health officials who have contributed critically to the research implementation and data analysis have been included as co-authors.

### Posionality statement

This paper has five Indigenous co-authors. CZ-C is a primary care physician of Quechua Indigenous heritage who has a PhD in health geography. CZ-C was born, in a Peruvian province called Ancash, and received her education in both Peru and Canada, supported by merit-based scholarships. As the principal investigator, she designed the study, supervised the collection and analysis of data, conceptualised the manuscript and contributed to its writing. VC-Z is a Peruvian female researcher with a Quechua background, and she supervised the collection and analysis of quantitative and qualitative data and contributed to the drafting of the first version this paper. IA-R is a Peruvian Quechua female researcher, and she provided key feedback for the ideation of the study and the analysis of the data collected. ED-L-C is an Ashaninka female and a leader in the OMIAASEC Indigenous organisation, who assisted the survey design, the application of the interviews and the implementation of the Junin workshop, translating when need it. She also provided relevant feedback for the analysis of the information. GL-R is a Shawi man and nurse technician currently affiliated with the Cayetano Heredia University and who was the main connection between the study and the Indigenous organisation, Gobierno Territorial Autónomo de la Nación Shawi. He assisted the application of the interviews, introduced the study objectives and methods to each of the Shawi communities and participants, and assisted the Loreto workshop, translating when necessary. He was also a speaker in the climate change and health course. All this team was part of a previous investigation conducted in these same regions, where we identified that improving the resilience of health systems to better serve Indigenous communities, was a priority for Peru, our home country.

## Results

### Are the health systems adapting and building resilience to climate change?

#### Key responses to climatic hazards reported

Responses were described according to six different dimensions of climate-resilient health systems: anticipate, respond, cope, recover, adapt and transform. To contextualise the responses reported by participants, we provided a characterisation of the climate hazards, exposure, vulnerability and health risks identified in this health system in Vidal-Cuellar *et al*.^44^

#### Anticipate

Official health systems took various measures to anticipate climate hazards and prevent health risks. First, disaster risk management offices established within the studied health networks delivered information on imminent climate events to health posts and communities to anticipate health assistance delivery. Interviewees reported that sometimes they could not access this information on time due to remoteness and poor connectivity, impairing their preparedness for climate events.

The risk office report extreme climate events, for example, ‘exceptional rains are coming’. They communicate us and all the health posts. They communicate with us and with the communities so that they take the event into consideration and are prevented, right? They communicate that exceptional rain is coming so they are on alert. And particularly if they have to transport an injured of emergency by river because the boats of transportation are small, and an accident can happen. (Official health system, health sector, Loreto region)

The information sometimes does not reach remote health facilities due to the internet connection issue. For example, if the current day very early, they send the risk management office information about a heavy rain, we share it, but sometimes it cannot be disseminated properly because connectivity problems. (Official health system, health sector, Junin region)

Second, health networks and health facilities trained communities in healthy practices to prevent climate-sensitive health risks. These practices included vaccination, keeping warm during cold waves, hand washing and proper food preparation and water treatment (ie, water boiling and chlorination). Finally, both health networks have established Environmental Health Offices, which are responsible for monitoring air and water quality and environmental factors that influence vector-borne diseases. On the other hand, Indigenous health systems used Indigenous knowledge to predict climate events through the observation of nature, helping to avoid climate-sensitive health risks. For instance, Indigenous knowledge of climate variability guided them to temporarily relocate themselves away from the river to avoided the impacts of floodings. Another measure was the use of Indigenous medicine to prevent climate-sensitive health risks. For example, before the windy season had started, Indigenous people used medicinal plants to protect themselves from acquiring airborne diseases.

At the beginning of August, the weather is changing so we have to be prepared. I take a sachawiro, portoncillo, ginger, beehive, and sugar cane, and I crush it and cook it. It is prepared like syrup, so you take it in a teaspoon, like syrup, that helps to treat the cold. (Indigenous health system, wise woman, Loreto region)

Finally, Indigenous communities by themselves or with the support of health posts, performed waste management and cleaning interventions in their communities. For instance, they cleaned the waste and threw out items that hold water to prevent vector-borne diseases, and cleaned water streams to reduce the risk of flooding. Despite our interviewees reporting the official health system taking anticipatory measures, they stressed these were not enough since the majority of responses focused attention on during and after the climate hazard.

Before the events, almost nothing is done… once the event happens, then everyone reacts, but the disaster is already done, the problem is nothing or little is done to prevent it. (Official health system, health sector, Junin region)

Regarding the flooding, there were problems in terms of preparation. No, we haven’t prepared ourselves properly, as if to say, ‘We can deal with such a situation’. (Official health system, health sector, Loreto region)

#### Respond

To respond to climate hazards, the official health systems in Loreto and Junin had intersectoral platforms that register climate emergencies and provide immediate health assistance. These platforms include representatives of the health networks, local governments, firefighters and the army. Health networks led emergency brigades to provide first aid and medical supplies, perform vaccination campaigns and evacuate individuals located in risky areas to temporary shelters. Also, health networks delivered water bottles and chlorinated the water tanks of health facilities and the populations affected, while local governments provided food or warm clothes if cold waves had started. Challenges for implementing these responses included limited financing to provide material resources and mobilise health brigades, and the unwillingness of some communities to leave risky areas.

As a challenge, I have always said: the budget. Because without money you do nothing. Sending a health brigade to an Indigenous area is very difficult. It is expensive, It is also difficult to find health workforce who want to go and are committed. This has been a weakness for us. (Official health system, health sector, Loreto region)

Finally, another response was the design of contingency plans by disaster risk management offices. The official health system implemented all these responses in coordination with Indigenous representatives since the latter could provide insights into community health needs and the populations affected, and help identify what kind of support was required. Indigenous peoples also took measures by themselves to respond to climate hazards. These included using mechanisms to evacuate affected areas based on their experience from previous climate disasters.

#### Cope

The official health system coped with climate hazards by performing arrangements in the organisation and distribution of the health workforce and medical products to maintain healthcare delivery. For instance, in the Loreto region, when flooding damaged the walls of the health posts, the health workforce was moved to a health facility located in an area that could not be reached by the water. If the capacity of the health posts was exceeded, the regional government or the Ministry of Health may provide support with additional resources. The Indigenous health system coped with climate hazards by implementing healthy practices, such as wearing warm clothes during cold waves and long-sleeved shirts during heat waves, and avoiding going to their farms during heavy rains. They also used Indigenous medicine to treat climate-sensitive diseases, namely skin injuries, snake bites and malaria, and used Indigenous practices to make climate hazards less dangerous (eg, using bows and arrows to reduce the impacts of strong winds on their communities).

We do not forget our traditions, when the winds come, the strong winds. We have to have faith, and put our arrows, bows, and ballistae, in the direction in which the wind is coming. Our ancestors have done it that way, right? Let’s say, this is my bow, and where does the wind come from? I plant my bow where the wind is blowing, so It can calm down. (Indigenous health system, community health agent, Junin region)

Finally, Indigenous people relied on the forests and rivers to obtain food when they have lost their crops due to climate hazards. They protected their food systems by hunting wild animals, gathering fruits and fishing. In some communities that are more used to monetary transactions, they used their savings to buy food from stores. The preference of Indigenous people for their own medicine and practices represented a challenge for health officers because the former may avoid using official healthcare services to treat climate-sensitive diseases.

The worldview of Indigenous peoples is that malaria can be treated with other types of medicines or preparations that are not what the official health system manages. Sometimes we struggle with that situation but it is in a minimal way. In general, the majority of people accept the official treatment given by the health establishments. (Official health system, health sector, Loreto region)

[When the health brigade visited the community after the health hazard], we did a haemoglobin test to see who was anaemic, and then we realised everyone was anaemic. And we left them sulfate. But the issue is we go and leave the medicine, but it’s not their own medicine, so we lack someone informing us which are the medicine and the food they prefer. (Official health system, health sector, Junin region)

#### Recover

After the occurrence of climate hazards, the official health systems provided material resources and humanitarian aid to support the recovery of affected communities. The local governments and the Civil Defense Institutions were responsible for delivering household materials (eg, cookware, roofs, blankets and tents), food or seeds and cash aid, according to local needs. Local governments were also in charge of restoring transportation access and infrastructure in damaged areas. However, our Indigenous participants reported these aids arrived late, were insufficient, did not arrive at all or could not be claimed because of connectivity gaps.

It arrives late, it is not instantly. There is flooding, and the food aid is not coming tomorrow or today. It arrives in one month or two months. (Indigenous health system, community leader, Loreto region)

If I don’t have a cell phone or something to take a photo for the evidence, how can they assist you? They are never going to assist you. ‘You must be lying’ they will say. It is with technology, and Ashaninka people do not know it, they’ve used a smartphone (Indigenous health system, community leader, Loreto region)

The State takes a long time to provide the aid, about 15-20 days to send the support. They have a certain bureaucracy. Until they send all the documentation, the reports, and the collection of information. (Official health system, private sector, Junin region)

Moreover, in some cases, the resources provided were not culturally appropriate for remote Indigenous communities, for instance, processed food, or suffocating clothes. Indigenous peoples recovered from climate hazards by organising themselves to clean and restore affected paths, community infrastructure and households. Furthermore, they shared or exchanged food and seeds in their communities to help each other. Since climate hazards generated crops and livestock losses, which were the main source of economic income for Indigenous families, and because the official aid is perceived as insufficient, this mechanism was considered an essential measure to support affected families in recovering from climate hazards.

We supported each other, we gave food, and if we had cassava or plantain we would share, so we could be able to resist, right? Until our crops would produce again. That is how we supported each other, we helped each other. (Indigenous health system, community health agent, Junin region)

#### Adapt

After the occurrence of climate hazards, the official health systems in Loreto and Junin have taken measures to recover their functions and take advantage of the situation in the long term. These measures were the development of climate change adaptation policies, community training strategies, promoting forest conservation, adapting health and household infrastructure and implementing health programmes targeted to climate-sensitive diseases. In 2021, policy roadmaps were developed by the Ministry of Health to guide the implementation of climate change adaptation measures in the Loreto and Junin regions by 2025. Each region had its own policy roadmap, has prioritised adaptation measures and indicators according to the local situation, and has defined the level of risk for each province and district by assessing the exposure, vulnerability and climate hazards. These measures were aligned with Peru’s National Adaptation Plan for Climate Change, also launched in 2021.^27^ Besides, both Loreto and Junin had Regional Strategies for Climate Change developed by regional governments, which contained adaptation and mitigation measures, and proposes coordination with public and private actors. Furthermore, the official health system has implemented community training strategies to improve the preparedness of vulnerable populations to respond to future climate emergencies. Forest conservation is another response taken to reduce the impacts of climate hazards on health in the long term. This was a response performed jointly by the official health system and the Indigenous health system in both Loreto and Junin. Indigenous peoples protected the forests by participating in reforestation projects developed along with forest conservation programmes led by Ministry of Environment and supported by NGOs, prohibiting logging, avoiding renting or using their lands for intensive agriculture, recovering the soil after intensive agriculture, legalising the property of their territories and adapting their economic activities to be less harmful to the forest.

The first thing we have done is to identify the eye of water that supplies the Indigenous community and the fish pond, so we have reforested that eye water. We have also articulated with other institutions to promote the reforestation (Official health system, NGO, Junin region)

We have worked with 15 communities, through a financial fund, planting yacushimbillo, a plant with lots of roots, that provides shade, protects the shore of the rivers and prevents landslides. (Official health system, local government, Loreto region)

We are conserving our forests because that is where our medicine is. There is all. Our market has always been there, It is related. (Indigenous health system, community health agent, Junin region)

Efforts to adapt healthcare and household infrastructure to climate conditions were also reported in both regions. In Loreto, there were intentions (not implemented yet) to maintain service delivery in the health posts even during flooding and rainy seasons. Moreover, in Satipo, the local government was working on developing household infrastructures resistant to cold waves. Finally, the studied health networks have developed health programmes targeted to climate-sensitive diseases, such as malaria. The approach included close coordination with Indigenous leaders and community health agents, local plans developed in joint with the community, and providing training, implements and incentives for community health agents to eradicate malaria.

On the other hand, Indigenous peoples adapted to climate change by conserving the forest, modifying their livelihoods according to climate conditions and incorporating healthy practices. Indigenous communities also adapted their livelihoods to better prepare themselves for climate hazards. As a learning outcome after experiencing flooding or landslides, they lived and cropped away from the rivers or places prone to climate events, built river defenses, modified the altitude of their houses and enlarged their boats for their transportation. Finally, they also have adopted healthy practices to protect themselves from climate hazards in the long term, such as washing their hands and keeping well-fed to maintain themselves healthy if another hazard comes.

#### Transform

Indigenous communities have been transforming their livelihoods to be better prepared to face climate and environmental shocks. With the support of NGOs and public agencies, Indigenous peoples were implementing agroforestry, garden projects, fish farms and chicken rearing, for selling and consuming, as an alternative to fishing and hunting.

The Indigenous community used to consume fish from the river, and today the fish have diminished due to environmental pollution, there isn’t much anymore. There are no longer fish so now they are breeding in their fish farms. (Indigenous health system, community health agent, Junin region)

The mayor is very concerned about how to support the families affected by the flooding. Maybe chicken rearing, because chickens grow very quickly, they can also make fish farms, or give food […] We are thinking about which solution can be faster to provide to the feeding of the affected families (Official health system, local government, Loreto region)

Within the community, they were also opting for modifying their crops to increase their resilience to climate variability and hazards, for example, changing their coffee plantations to pineapple.

what crop to produce? For example, we used to crop coffee, but because of the climate variability it is not longer suited. So, what is the alternative for our income? Pineapple and raising smaller animals. So that’s what we’re doing, because of climate change (Indigenous health system, community health agent, Junin region)

#### The process of building health systems resilience and informing building blocks

**Figure 3:**
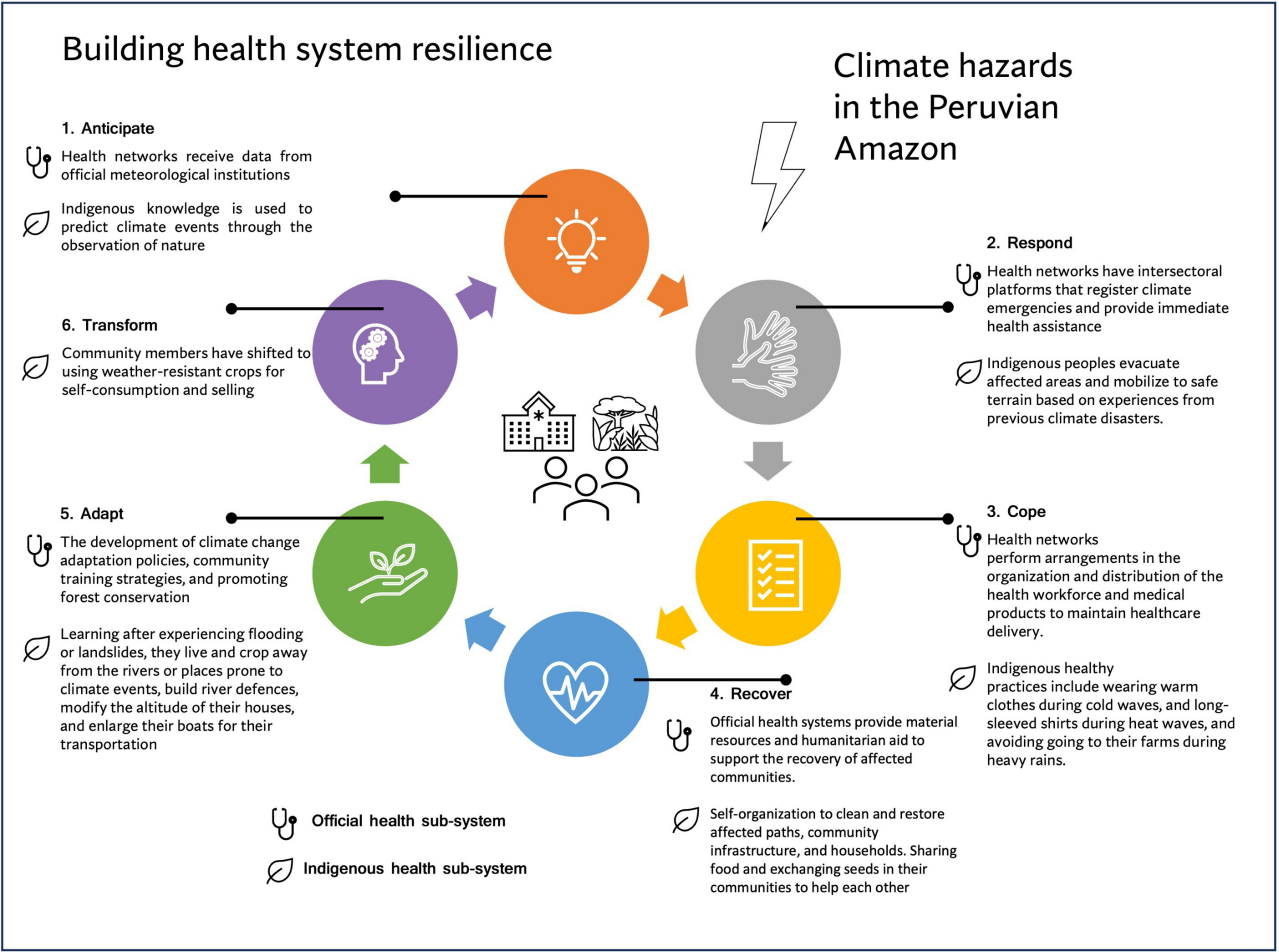
The process of building health system resilience in this Amazon Peruvian health system.

We found that both health systems (the Indigenous and non-Indigenous) were active on responding and adapting to climate hazards; however, only Indigenous responses were identified in the dimension of *transformation*. Since these responses were implemented on a temporal scale from anticipation of the climatic hazard to transformation after the hazard impact, figure 3 illustrates how the process of building health system resilience in this Amazon Peruvian health system was informed for the six response dimensions. We also found that responses from both official and Indigenous health systems have informed different building blocks of the health system (see table 1 in online supplemental material 5). In leadership and governance, Indigenous organisations were named as part of the climate change governance platform but only at the national level, with still little or non-participation at the district level or community level. For emergency preparedness, Indigenous responses were mostly oriented to prevent and anticipate, while responses in the official health system were for management once the climate hazards had already occurred. Attention to key environmental determinants of health differed across responses: the official health system was more oriented to providing safe air and water, while Indigenous responses were focused on protecting forests and cleaning the communities. For climate-informed health programmes and vulnerability and adaptation assessment, information indicated that official health systems had trained communities on the management of climate-sensitive diseases, although adaptation assessments were not yet performed. Indigenous and non-Indigenous health systems have used their own knowledge and information to predict climate hazards at community level, while research on health and climate was reported as a need to inform the implementation of adaptations in each region. Loreto and Junin recognised the importance of researching and implementing new technologies that reduce greenhouse gas emissions, while Indigenous technologies in their health systems were essentially based on the use of nature resources and sharing cultural practices.

### Key pathways to strengthen the climate resilience of health systems

This section of the results aimed to provide recommendations to increase the climate resilience of health systems in the Peruvian Amazon. We identified resilience pathways according to the WHO health systems building blocks. This section was prepared predominantly on the recommendations of the in-person workshops complemented with key findings included in Appendix A and B.^44 45^

#### Leadership and governance

Participants highlighted that strengthening leadership and governance of health systems in the face of climate change requires three main actions. First, it was proposed to enhance Indigenous representation in intersectoral governance platforms linked to climate change and health, mainly local governments, and disaster risk management platforms. Although regional health networks already have Indigenous offices to work jointly with Indigenous representatives and communities, there is still a need to strengthen the participation of Indigenous leaders in decision-making spaces. Proposed mechanisms to achieve this included establishing in-person dialogue spaces between Indigenous leaders and local decision-makers and formalising partnerships between Indigenous organisations and local authorities. Local Indigenous organisations could strengthen health and climate governance by providing insight into community health needs, vulnerabilities and capacity before and after climate events. Also, Indigenous knowledge regarding weather variability and climate hazards would help local governments take preventive measures. Besides, although the Peruvian government has created a platform formed by national Indigenous organisations to cope with climate change,^45^ there is still a need to strengthen communication channels between Indigenous local organisations and national and regional governance platforms to ensure that remote communities receive governmental support and humanitarian aid before and after climate disasters. Second, it was proposed to promote Indigenous women’s participation in the development of the climate change and health agenda at the local level. Since women were identified as vulnerable to climate change in both regions, participants in the workshops highlighted the need to engage women in regular meetings with local governance platforms to identify and assess women’s specific health needs in the climate change context. Indigenous women were recognised as the primary care takers before, during and after an extreme climatic event. Besides, it was noted that women’s participation requires to be enhanced not only at the policy level but also at the community level by recognising women’s role in food governance and food security interventions. Participants stressed that these interventions need the support of health and other health-determining sectors at the governmental level to ensure they are feasible and constant over time. Finally, strengthening cross-sectional collaboration between the health, environment and agriculture sectors was also recommended as a pathway to increase the climate resilience of health systems. In both regions, participants reported that there is a lack of articulated policies and strategies involving both the health and the environmental sectors to address the impacts of climate change on health. This is particularly important to address food insecurity and biodiversity loss, which were identified as key climate-sensitive health risks in both regions. Actions proposed to achieve this goal were mainly to strengthen local governments’ leadership in disaster risk management platforms and improve articulation with private enterprises involved in environmental management.

#### Health workforce

Building trust with Indigenous communities is one of the key challenges experienced by the health workforce in the Peruvian Amazon, and it becomes particularly important in the context of climate change, in which coordinated action is needed. For example, in the survey from 13 health facilities visited, all of them reported the need to coordinate with community health workers for health emergencies, however, only 3 (23%), coordinated with Indigenous healers such as ‘curandero’, ‘partera’, ‘sobador’ and ‘vaporera’. Participants reported that they did not trust Indigenous healers because they did not meet them before, did not know how Indigenous medicine works or Indigenous healers did not trust on the health sector either. Given that Indigenous healers are the custodians of knowledge about Indigenous medicine, there is a lost opportunity to articulate the Indigenous and non-Indigenous systems if this relationship is not improved. Moreover, in the survey, only two health facilities (15%) had at least one health worker trained on intercultural health approaches and three (23%) had health workers that spoke an Indigenous language, reflecting the gap of knowledge about Indigenous people’s culture and medicine that persist in the Peruvian health work force. A potential mechanism proposed to strengthen this relationship is to incorporate Indigenous health workers in health centres and health posts, as this would increase the acceptability of health interventions as well as the utilisation of health services by Indigenous peoples. Formally incorporating Indigenous health workers would help improve the organisational capacity of Amazon health systems to cope with climate change, and would also enhance the ability of official health systems to partner with community members. Another recommendation relevant to this building block was to develop training interventions on climate change and health addressed to official authorities and disaster risk management platforms. These recommendations could help local health systems and other health-determining sectors develop communication and awareness-raising strategies on climate change and health. Participants highlighted the need to raise awareness among private enterprises that contribute to deforestation and water contamination. Also, participants proposed to develop awareness-raising interventions on climate change and health targeted to Indigenous communities. Some actions suggested for achieving this goal include creating radio programmes for remote communities and designing visual materials on climate change and health culturally appropriate for Amazon Indigenous peoples. This latter measure would facilitate reaching out to illiterate Indigenous adults and young children. Finally, it was recommended to increase the number of specialists in healthcare facilities located in remote areas, mainly mental health professionals, dermatologists and nutritionists. In our study, only 1 out of the 13 health facilities surveyed reported having nutritionists (a hospital located near the main urban centre of the Junin region). Increasing the number of medical specialists is considered important to manage specific climate-sensitive health risks, namely, mental health outcomes resulting from experienced climate disasters, skin lesions produced by extreme temperatures, and severe malnutrition cases resulting from food insecurity and biodiversity loss. Only 1 out of 13 (8%) health facilities reported to have a nutritionist and 4 (31%) did not have a any type of physician to provide healthcare in situ.

#### Health information systems

Participants emphasised that strengthening health information systems requires creating mechanisms to share local data on climate change and health vulnerability assessments with communities. This pathway would help raise awareness among communities about their current and projected situation in the face of climate change, thereby facilitating community engagement in disaster risk management. Moreover, this pathway also emphasises the importance of enhance the communication for health emergencies, since only 3 (23%) out of 13 health facilities reported to have any type of communication systems working all day, and 4 (31%) had access to internet.^44^ Another pathway proposed to improve health information systems was to strengthen the scientific evidence on the use of Indigenous medicine and Amazonian foods for managing climate-sensitive health risks. Incorporating this topic into the research agenda in the Peruvian Amazon could provide insight into local solutions and capacities and make scientific evidence relevant to local contexts. Also, considering Indigenous health practices and priorities would open opportunities to establish transdisciplinary teams in the health research field. In our cross-sectional survey, only 3 out of 13 health facilities reported coordinating with Indigenous medicine agents for health interventions.

#### Service delivery

To strengthen health service delivery in a climate change context, participants proposed to expand the scope of health promotion interventions by encouraging environmental protection practices (mainly forest protection and care of natural water sources) as healthy behaviours among Indigenous communities. This pathway was recognised to be particularly relevant for local communities since Indigenous leaders reported that some community members still adopt behaviours that perpetuate environmental hazards due to multiple factors (eg, deforestation practices reinforced by economic needs). In this context, the official health system could play a key role in raising awareness of the health benefits of environmental protection practices and contributing to joint monitoring of environmental exposures and outcomes along with other health-determining sectors. Under the WHO operational framework, this pathway would contribute to the management of environmental determinants of health and help to promote a ‘Health in all policies’ approach by engaging affected communities.^22^ Other actions suggested for managing environmental determinants of health included implementing recycling projects to improve solid waste management in remote communities to reduce mosquito breeding sites. We observed that 10 (77%) out of the 13 health facilities surveyed in Loreto and Junin burn and/or bury common waste regularly, and 7 (54%) have access to a municipal garbage service. Another action proposed was to create a community-based monitoring system to supervise river cleaning and water quality in remote communities. All these actions require not only the participation of health-determining sectors at the policy at programmatic levels but also active coordination and engagement with communities.

Finally, it was also proposed to strengthen the training of CHAs and equip them with medical products to respond to climate disasters. CHAs in Loreto and Junin were reported to have a key role in delivering health promotion information to Indigenous communities.

#### Essential medical products and technologies

The first pathway proposed to strengthen this building block was to ensure access to water and sanitation in healthcare facilities located in remote communities. Data from our cross-sectional survey showed that 11 (85%) out of 13 health facilities have no access to drinkable water, 7 (54%) have no or no functioning showers and 4 (31%) lack a water supply for hand washing. Improving access to water and sanitation would require the participation of local governments and health systems to plan investments in the construction and improvement of healthcare facilities according to current and projected climate risks. Second, since food insecurity and biodiversity loss were recognised as key climate-sensitive health risks for Indigenous peoples, another relevant pathway proposed was to promote the participation of Indigenous communities in the use and implementation of new technologies to strengthen food security systems. These technologies may include fish farms, organic gardens, bird breeding projects and the implementation of climate-resilient agriculture practices according to local climate conditions. Although the implementation of food adaptation technologies is usually managed outside the conventional health sector, strengthening the articulation with the health sector for these interventions could help reach out to remote communities and facilitate resource mobilisation. For example, health promotion offices of the regional health networks regularly perform demonstrative sessions, in which the health workforce teaches how to prepare healthy foods with the support of these new technologies. Besides, this pathway would strengthen the service delivery component by contributing to managing the environmental determinants of health.

#### Financing

**Figure 4:**
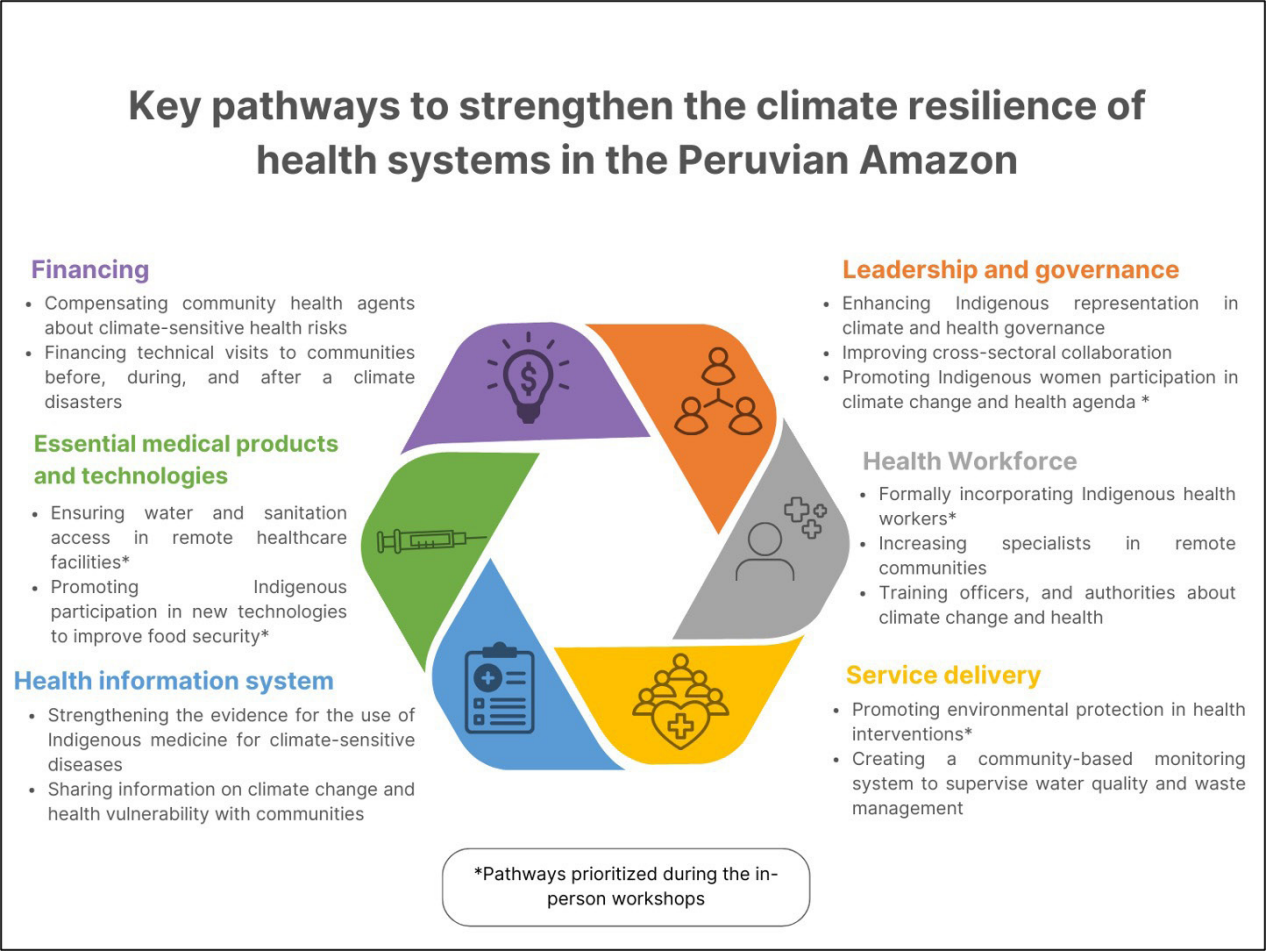
Key pathways to strengthen the climate resilience of health systems in the Peruvian Amazon.

One of the most relevant financing gaps identified is the lack of financial support for CHAs, considering their key role in supporting the prevention and control of climate-sensitive health risks. This gap affects various other health system building blocks as it exacerbates CHAs’ workload and impairs their engagement with the official health system. For instance, in Loreto, it was reported that CHAs are changed every 1–3 years due to this reason, affecting the coordination and implementation of health promotion interventions in communities in the mid and long term. Another recommendation identified in this building block was to increase the budget of local governments and health systems to enable health workers and disaster risk management officers to perform technical visits to remote communities before, during and after a climate disaster. Before climate events, these visits would help ensure that community households are located in safe zones, and also deliver education interventions about climate and health risks (eg, implementing emergency and evacuation drills and developing emergency protocols). During and after the events, facilitating visits to remote communities would help provide them with material resources and humanitarian aid to ensure their recovery and manage adverse health outcomes. Figure 4 shows the each building blocks pathways to strengthen the climate resilience of health systems in the Peruvian Amazon,

## Discussion and conclusion

This study addresses the gap in the literature on the climate change resilience of health systems with a focus in the Peruvian Amazon health systems by working with Indigenous leaders, community members and health officers. We found that the Amazon health system was adapting to climate change. We identified a series of Indigenous and non-Indigenous responses contributing to the adaptation and resilience to climate change. Indigenous responses to climate hazards included the use of Indigenous knowledge on weather variability, Indigenous medicine to manage health risks and community networks to share food products and resources. The official health systems in both Loreto and Junin have developed regional strategies for climate change and response platforms that act mainly after the occurrence of climate disasters. Key challenges identified to implement climate change health responses included lack of financing for climate change actions, limited scientific evidence on climate change impacts on health and absence of coordination across different sectors besides health. We also found that multiple Indigenous responses were not yet articulated within the official health systems. For example, addressing the environmental determinants of health in the health sector was focused on controlling specific diseases like vector borne disease, while Indigenous practices were reported to protect the forest and natural environment as a whole system.

We propose that key resilience pathways identified in this study, call for a broader paradigm shift in health systems, that recognises Indigenous resilience as valuable for health adaptation, moves towards a participatory health system and broadens the vision of health as a dimension inherently tied to the environment. In this study as in other settings, Indigenous responses have proven to be crucial for health adaptation to climate change, with the potential to bring insights for decision-making and the co-production of knowledge for better adaptation measures.^1546^,^49^ Recognising Indigenous responses as valuable requires further characterising local Indigenous responses to climate hazards and strengthening the cultural competency of the health workforce to acknowledge both Indigenous and scientific knowledge systems as equally valid, relevant and useful. Previous case studies suggest that combining Indigenous and official health systems’ knowledge and responses to climate change may open opportunities for bidirectional learning, and a better understanding of links between climate change impacts and responses to protect health.^850^,^52^

Moreover, the recognition of the relevance of Indigenous responses must lead to the transition to a participatory model of healthcare delivery, with Indigenous communities and other underserved populations being affirmed as partners in health governance, rather than just recipients of care.^53^ The implementation of participatory healthcare models that enabled community members to take ownership of their health and well-being has been shown to improve health outcomes significantly.^54^,^56^ In a climate change context, community engagement is key as it provides an entry point to strengthen social capital, greater adaptability to emerging challenges, and therefore, increased resilience at the individual and community levels.^1957^,^60^ The articulation of Indigenous responses into the health system could be enriched with a holistic approach recognising the inherent connection between health and the natural environment. Incorporating such a holistic understanding requires government support, societal participation and capacity building.^61 62^

We aligned our climate resilience pathways with the WHO health system building blocks, and this framework allowed us to acknowledge the different components of the health systems, which strengthened our understanding of how climate change affects health systems across different functionalities (health workforce, financing, governance, etc). To improve the preparedness of health systems for climate change impacts in multicultural settings, we recommend that people’s lived experiences on the ground to be considered when using this framework. To adapt the building blocks to local contexts and increase the social value of health interventions for frontline communities, we would recommend integrating the following three dimensions across all the building blocks: (1) the role of community members and organisations in providing inputs for local decision-making, (2) the value of culturally different understandings of health and health sub-systems in the climate change context and (3) gender roles in implementing healthcare practices for individuals, families and communities before, during and after climate hazards.

### Limitations and strengths

Our study was conducted in close collaboration with Indigenous organisations and involved the active participation of Indigenous researchers. However, we recognise that the framing of our methodology was influenced by mainstream scientific perspectives on health. Moving forward, we aim to explore Indigenous knowledge regarding responses to climate change and the concept of health system resilience from Indigenous cosmogonies. Despite this limitation, we employed a mixed-method design, enabling us to triangulate our results and thereby provide stronger evidence, particularly concerning the interconnection between Indigenous and non-Indigenous health systems. Our methodology has the potential to be replicated in similar multicultural contexts beyond the Peruvian Amazon.

## Supplementary material

10.1136/bmjgh-2023-014391 online supplemental file 1

10.1136/bmjgh-2023-014391 online supplemental file 2

10.1136/bmjgh-2023-014391 online supplemental file 3

10.1136/bmjgh-2023-014391 online supplemental file 4

10.1136/bmjgh-2023-014391 online supplemental file 5

10.1136/bmjgh-2023-014391 Abstract translation 1 This web only file has been produced by the BMJ Publishing Group from an electronic file supplied by the author(s) and has not been edited for content.

## Data Availability

Data are available upon reasonable request.
