# Supplementary material for: Pathways to strengthen the climate resilience of health systems in the Peruvian Amazon by working with Indigenous leaders, communities and health officers
Source: BMJ Glob Health. 2024 Sep 7;8(Suppl 3):e014391. doi: 10.1136/bmjgh-2023-014391 (PMC11733073; doi:10.1136/bmjgh-2023-014391)
Supplement: online supplemental file 1 [file bmjgh-8-Suppl_3-s001.pdf]

Supplementary Material 2

1. Interview guide to official health systems (spanish)

Guía de preguntas para entrevista semi estructurada para personal de salud y de otros sectores, sobre peligros climáticos a la salud, vulnerabilidades y respuestas en la región Junín y Loreto

Objetivo: Caracterizar el sistema de salud, su capacidad de respuesta y de adaptación a eventos climáticos (o peligros), y cómo se articula con la respuesta de las poblaciones Indígenas

Participantes: Personal de salud y otros sectores relevantes que gestionan la respuesta y adaptación al cambio climático, mayores de 18 años, localizados en la región de Junín y Loreto

Antes de iniciar se debe corroborar que el participante este cómodo, y haya brindado su consentimiento para ser grabado o para tomar notas. Las peguntas se aplicarán en español. Se grabará sólo si el participante lo autoriza.

**Tema A: Los eventos climáticos y la salud**<br>**Descripción: Identificar los peligros climáticos para la salud desde la perspectiva del personal de salud y otros actores locales**
1. Podría comentarnos por favor: su edad, a que se dedica (qué cargo tiene en su institución)<br>2. ¿Cuál es la función principal de la institución donde usted trabaja?<br>3. Su organización atiende a población Indígena, Si, es si.<br>3.1. Aproximadamente ¿qué porcentaje de la población que está bajo la responsabilidad de jurisdicción, es población o comunidades Indígenas?<br>4. ¿Cómo el medioambiente afecta la salud de las familias de esta zona, su salud, y la de su familia?<br>5. ¿Diría que en los últimos años ha habido cambios en las estaciones en su zona? Si, No Si, si<br>6. ¿Puede explicarnos cuáles cambios en las estaciones? (si no menciona puede leer: aumento de temperatura, inundaciones más frecuentes, vientos más intensos)<br>6.1. ¿Desde cuándo nota estos cambios?<br>6.2. ¿A quiénes o a que, afecta más estos cambios? ¿Y cómo así?<br>6.3. En su institución, ¿han registrado o reportado esos cambios?<br>7. ¿Cuál característica del clima de su zona, diría usted que es un peligro importante para la salud de su población, ¿Y por qué?<br>7.1. ¿A quiénes o a que sectores, afecta más estos peligros? ¿Y cómo así?<br>8. ¿Existen otros peligros para la salud de usted, de su familia o de su población que desee mencionarnos?<br>9. ¿qué entiende por **adaptación** al cambio climático?<br>10. ¿qué entiende por **mitigación** al cambio climático?<br>11. ¿Has escuchado sobre **resiliencia** al cambio climático? si, no . Si es si, ¿nos puede comentar lo que ha escuchado?<br>12. ¿Actualmente su organización tiene algún plan de adaptación para peligros relacionados al cambio climático? Si,____anotar el nombre del plan____<br>No, pero estamos actualmente preparando uno y estará listo en el siguiente año<br>No, pero estamos planeando uno en los siguientes dos años<br>No, no he escuchado de esto

No, no sé al respecto

13. ¿Actualmente su comunidad (u organización) tiene algún plan para **prevenir** los impactos al cambio climático?

Si,____anotar el nombre del plan

No, pero estamos actualmente preparando uno y estará listo en el siguiente año

No, pero estamos planeando uno en los siguientes dos años

No, no he escuchado de esto

No, no sé al respecto

Tema B: Eventos climáticos del pasado (peligros) y como se enfrentaron
Descripción: Explorar la memoria, respuesta y aprendizaje en relación con un peligro climático en el pasado (en esta sección solo se aplicarán las preguntas o del bloque A o del bloque B)

Bloque A

10. ¿Podría recordar un evento extremo relacionado al clima que ocurrió en el pasado en su zona?

Un evento extremo relacionado al clima es un evento único que genera pérdidas materiales y/o humanas que se ocasiona por una situación inusual en el clima. Por ejemplo, en base a su región, sabemos que algunas veces las lluvias se incrementan y causan que se desborde el río. También puede ser por una presencia anormal de una ola de frio que afecta a la población. Otro evento puede ser que la temperatura del ambiente es muy alta, y la población se ve afectada

Las siguientes preguntas serán en base al evento que ha recordado, o en caso no recuerde un evento climático, nosotras le mencionaremos un evento en el pasado que ha ocurrido cerca de su zona.

(Si aun así no es posible que el participante recuerde la información, le pediremos que nos responda en base a la experiencia del COVID-19 -usar las preguntas del bloque B)

10.1. Puede comentarnos ¿qué fue lo que ocurrió? Y en que fecha aproximadamente

10.2. ¿Según su conocimiento o percepción, qué lo puede haber originado?

10.3. ¿Qué o quienes resultaron más afectados? (pueden ser infraestructura, familias, cultivos, niños, adultos, gestantes, ancianos, etc)
¿Alguien falleció?

10.4. ¿Cuánto tiempo duró?

10.5. ¿Recuerda que hizo la población para protegerse del evento?

10.5.1. ¿De dónde sacaban agua para beber?

10.5.2. ¿Cómo hacían para alimentarse?

10.5.3. ¿Como hacían para ir a trabajar (a su chacra, o a su oficina, o a su colegio)?

10.6. ¿Recuerda que hizo su establecimiento de salud (o su organización), para proteger a la comunidad del evento (antes si se tenían planes de preparación), durante o después? ¿Nos puede dar un ejemplo de lo que hizo?

10.7. ¿Recuerda que hizo su establecimiento de salud (o su organización), para proteger a sus trabajadores del evento, (antes, si tenían algún plan de preparación), durante o después? ¿Nos puede dar un ejemplo de lo que hizo?

10.8. ¿Coordinaron con otros sectores diferentes de salud (diferentes de su organización) , durante o después del evento? ¿Si fue así, nos puede dar un ejemplo de esa coordinación?

10.9. ¿Existió alguna coordinación con la comunidad o lideres indígenas, antes, durante el evento, o después del evento? ¿Si fue así, nos puede dar un ejemplo de esa coordinación?

10.10. ¿Cuánto tiempo diría usted que le tomó a su establecimiento de salud (o su organización), volver a

la normalidad, después que terminó el evento?

10.11. ¿Podría mencionar algún aprendizaje que le dejó el vivir este evento que podría ayudarle en el futuro a cómo funciona su establecimiento de salud (su organización)?

Bloque B: *Si comenta sobre COVID:*

10.12. ¿Qué o quienes resultaron más afectados? ¿Alguien falleció?

10.13. ¿Diría que actualmente ya volvieron a normalidad?

10.14. ¿Recuerda que hizo la población para protegerse en los momentos más críticos de la pandemia? Por ejemplo, de donde sacaban agua para beber, cómo hacían para alimentarse, como hacían para ir a trabajar (a su chacra, o a su oficina, o a su colegio)?

10.15. ¿Recuerda que hizo su establecimiento de salud (o su organización), para proteger a la comunidad del
evento (antes si se tenían planes de preparación) , durante o después? ¿Nos puede dar un ejemplo de lo que hizo?

10.16. ¿Recuerda que hizo su establecimiento de salud (o su organización), para proteger a sus trabajadores del evento, (antes, si tenían algún plan de preparación), durante o después? ¿Nos puede dar un ejemplo de lo que hizo?

10.17. ¿Coordinaron con otros sectores diferentes de salud (diferentes de su organización) , durante o después del evento? ¿Si fue así, nos puede dar un ejemplo de esa coordinación?

10.18. ¿Existió alguna coordinación con la comunidad o lideres indígenas, antes, durante el evento, o después del evento? ¿Si fue así, nos puede dar un ejemplo de esa coordinación?

10.19. ¿Podría mencionar algún aprendizaje que le dejó el vivir este evento que podría ayudarle en el futuro
a cómo funciona su **establecimiento de salud** (su organización)?

Tema C: Articulación del sistema de salud Indígena y salud oficial, para responder de un peligro climático
Descripción: Identificar espacios donde se podría mejorar la articulación del sistema de salud Indígena y el occidental

11. ¿Actualmente, existe algún tipo de coordinación entre su establecimiento de salud (o su organización) y las comunidades Indígenas para proteger la salud de las personas en momentos de un evento climático extremo?
12. ¿Cuáles cree que son los desafíos para articular mejor las acciones de su establecimiento de salud (o su organización) y las comunidades Indígenas para proteger la salud de las personas en momentos de un evento climático extremo?
13. ¿Cómo cree que se podría articular mejor las acciones de su establecimiento de salud (o su organización) y las comunidades Indígenas para proteger la salud de las personas en momentos de un evento climático extremo?
    13.1. ¿Puede mencionar al menos dos sugerencias para mejorar la articulación entre su organización y las comunidades Indígenas? (si no responde, se le puede mencionar: en términos de información, coordinación, liderazgo, financiamiento)
14. ¿Cómo cree que se podría articular mejor las acciones de su establecimiento de salud (o su organización) con otros sectores (educación, ambiente, cultura), para proteger la salud de las personas en momentos de un evento climático extremo?

14.1. ¿Puede mencionarnos al menos dos sugerencias para mejorar la articulación entre su organización y otros sectores? (si no responde, se le puede mencionar: en términos de información, coordinación, liderazgo, financiamiento)

2. Interview guide to Indigenous health systems (spanish)

Guía de preguntas para entrevista semi estructurada con sabios, sabias y agentes comunitarios Indígenas

Objetivo: Caracterizar el sistema de salud, su capacidad de respuesta y de adaptación a eventos climáticos (o peligros), y cómo se articula con la respuesta de las poblaciones Indígenas

Participantes: Personas mayores de 18 años, que se autoidentifican como Indígenas, pueden ser líderes, trabajadores comunitarios de salud, promotores de salud, médicos tradicionales, parteras u otro sabio y sabia Indígena relacionada ala salud de la comunidad.

Antes de iniciar se debe corroborar que el participante este cómodo, y haya brindado su consentimiento para ser grabado o para tomar notas. Las preguntas se aplicarán en español e idioma indígena. Se grabará sólo si el participante loautoriza.

**Tema A: Caracterización del sistema de salud Indígena**<br>**Descripción: Lograr comprender las piedras angulares del sistema de salud indígena: 1) liderazgo, 2) actores claves que brindan atención, 3) cómo se transmite la información sobre salud y enfermedad, 4) qué recursos se usan para poder brindar las atenciones, y donde se brindan 5) que condiciones de enfermedad son las que predominan 6) como se financian estas atenciones**
1. Podría comentarnos por favor ¿, su edad, a que se dedica, el nombre de su comunidad?<br>2. ¿Cumple algún rol de liderazgo en su comunidad? (por ejemplo, en la junta vecinal, en el colegio, el vaso de leche)<br>3. ¿Qué es salud para usted?<br>4. ¿Qué enfermedades o males son los más frecuentes en su comunidad? (listarlos)<br>5. De estos que mencionó ¿Cuál diría usted que son las tres más importantes y deben ser atendidas como prioridad? Y ¿por qué?<br>6. Las siguientes preguntas son en relación a una de las condiciones que el participante mencionó como prioridad (pedir al participante que elija una que desee comentar):<br>6.1. ¿A dónde acuden los comuneros y comuneras cuando tienen_____?<br>6.2. ¿Quién se encarga de tomar las decisiones cuando alguien se enferma con_____?<br>6.3. ¿Quien o quienes son los encargados de brindar la atención cuando alguien se enferma o se siente mal con _______________?
6.4. ¿Qué medicinas u otros recursos, se usan para sanarse de esta condición________?<br>6.5. ¿Quién o quiénes se encargan de transmitir información sobre estar sanos, o para prevenirla?<br>6.6. ¿Qué tipo de costo (ya sea en tiempo, dinero u otro), debe asumir el paciente para poder atenderse cuando tiene____?<br>6.7. ¿Qué tipo de costo (ya sea en tiempo, dinero u otro) asume el que brinda la atención de salud_____?<br>7. Las siguientes preguntas son acerca de su relación con los servicios oficiales de salud<br>7.1. Me podría contar un poco acerca de la última vez que usted se atendió en el centro de salud (para que fue, como fue la atención, como accedió a medicamentos, etc.)<br>7.2. En tu comunidad, ¿para qué enfermedades los comuneros van a atenderse en el puesto de salud?<br>7.3. ¿Cómo considera que es la atención en el puesto de salud más cercano?<br>7.4. ¿Qué tanto confían los comuneros en el personal del puesto de salud?<br>7.5. ¿Cuáles cree que deberían ser las características o cualidades del personal de salud?

7.6. Si pudiera dar una sugerencia para mejorar su puesto de salud, ¿cuál sería?

7.7. Si usted fuera el jefe del puesto de salud y quisiera lograr que todos los comuneros se atienden ahí, ¿cuál sería su estrategia?

Tema B: Los eventos climáticos y la salud
Descripción: Identificar los peligros climáticos para la salud desde la perspectiva Indígena

7. ¿Cómo el medioambiente afecta su salud, y la de su familia en esta zona?
8. ¿Diría que en los últimos años ha habido cambios en las estaciones? Si, No
   Si, si
   8.1. ¿Puede explicarnos cuales cambios? (si no menciona leer para darle pautas: aumento de temperatura, inundaciones más frecuentes, vientos más intensos)
   8.2. ¿Desde cuándo nota estos cambios?
   8.3. ¿A quiénes o a que, afecta más estos cambios? ¿Y cómo así?
9. ¿Cuál característica del clima de su zona, diría usted que podría ser un peligro importante para la salud de su población, ¿Y por qué?
   9.1. ¿A quiénes o a que sectores, afecta más estos peligros? ¿Y cómo así?
10. ¿Existen otros peligros para la salud de usted, de su familia o de sus vecinos que desee mencionarnos?
11. ¿qué entiende por **adaptación** al cambio climático?
12. ¿qué entiende por **mitigación** al cambio climático?
13. ¿Has escuchado sobre **resiliencia** al cambio climático? si, no . Si es si, ¿nos puede comentar lo que ha escuchado?

14. ¿Actualmente su comunidad (u organización) tiene algún plan de **adaptación** para peligros relacionados al cambio climático? Si,_____anotar el nombre del plan
    - No, pero estamos actualmente preparando uno y estará listo en el siguiente año
    - No, pero estamos planeando uno en los siguientes dos años
    - No, no he escuchado de esto
    - No, no sé

1. ¿Actualmente su comunidad (u organización) tiene algún plan para **prevenir** los impactos al cambio climático? Si,____anotar el nombre del plan

- No, pero estamos actualmente preparando uno y estará listo en el siguiente año
- No, pero estamos planeando uno en los siguientes dos años
- No, no he escuchado de esto
- No, no sé al respecto

Tema C: Eventos climáticos del pasado (peligros) y como se enfrentaron
Descripción: Explorar la memoria, respuesta y aprendizaje en relación con un peligro climático en el pasado (enesta sección solo se aplicarán las preguntas o del bloque A o del bloque B)

Bloque A

12. ¿Podría recordar un evento extremo importante relacionado al clima que ocurrió en el pasado en su comunidad?*Un evento extremo relacionado al clima es un evento único que genera pérdidas materiales y/o humanas quese ocasiona por una situación inusual en el clima. Por ejemplo, en base a su región, sabemos que algunas*

veces las lluvias se incrementan y causan que se desborde el río. También puede ser por una presencia anormal de una ola de frio que afecta a la población. Otro evento puede ser que la temperatura del ambientees muy alta, y la población se ve afectada

Las siguientes preguntas serán en base al evento que ha recordado, o en caso no recuerde un evento climático, nosotras le mencionaremos un evento en el pasado que ha ocurrido cerca de su zona.

(Si aun así no es posible que el participante recuerde la información, le pediremos que nos responda en base a la experiencia del COVID-19 -usar las preguntas del bloque B)

12.1. Puede comentarnos ¿qué fue lo que ocurrió? Y en qué fecha aproximadamente

12.2. ¿Según su conocimiento o percepción, qué lo puede haber originado?

12.3. ¿Qué o quienes resultaron más afectados? (pueden ser infraestructura, familias, cultivos, niños, adultos, etc)

¿Alguien falleció?

12.4. ¿Cuánto tiempo duró?

12.5. ¿Recuerda que hizo la población para protegerse del evento?

12.5.1. ¿De dónde sacaban agua para beber?

12.5.2. ¿Cómo hacían para alimentarse?

12.5.3. ¿Como hacían para ir a trabajar (a su chacra, o a su oficina, o a su colegio)?

12.6. ¿Existió algún tipo de coordinación para proteger la salud de las personas de su comunidad, con el personal de salud, el centro de salud o el hospital? ¿Puede darnos un ejemplo de cómo fue la coordinación?

12.7. ¿A parte del sector salud, coordinaron con otros sectores del estado, durante o después de ese evento para proteger la salud de su comunidad? ¿Si fue así, cuanto tiempo después y como fue esa coordinación?

12.8. ¿A parte del sector salud, y los sectores del estado, coordinaron con otras comunidades indígenas, o lideres indígenas, durante o después de ese evento para proteger la salud de su comunidad? ¿Si fue así, cuanto tiempo después y como fue esa coordinación?

12.9. ¿Cuánto tiempo diría usted que les tomó volver a la normalidad, después que terminó el evento?

12.10. ¿Podría mencionar algún aprendizaje que le dejó el vivir este evento que podría ayudarle en el futuro a cómo funciona su comunidad?

Bloque B: *Si comenta sobre COVID:*

12.11. ¿Qué o quienes resultaron más afectados? ¿Alguien falleció?

12.12. ¿Diría que actualmente ya volvieron a normalidad

12.13. ¿Recuerda que hizo la población para protegerse en los momentos más críticos de la pandemia? Por ejemplo, de donde sacaban agua para beber, ¿cómo hacían para alimentarse, como hacían para ir a trabajar (a su chacra, o a su oficina, o a su colegio)?

12.14. ¿Recuerda que hizo el sector salud, para proteger a la comunidad del evento (antes si se tenían planes de preparación) , durante o después? ¿Nos puede dar un ejemplo de lo que hizo?

12.15. ¿Coordinaron con otros sectores diferentes de salud, durante o después del evento? ¿Si fue así, nos puede dar un ejemplo de esa coordinación?

12.16. ¿Existió alguna coordinación con la comunidad o lideres indígenas, antes, durante el evento, o después del evento? ¿Si fue así, nos puede dar un ejemplo de esa coordinación?

12.17. ¿Podría mencionar algún aprendizaje que le dejó el vivir este evento que podría ayudarle en el futuro a cómo funciona su **comunidad (o** su organización)?

Tema D: Articulación del sistema de salud Indígena y salud oficial, para responder de un peligro climático Descripción: Identificar espacios donde se podría mejorar la articulación del sistema de salud Indígena y el occidental

13. ¿Actualmente, existe algún tipo de coordinación entre las comunidades Indígenas y el sector salud, para proteger la salud de las personas en momentos de un evento climático extremo?
    - 13.1. ¿Cuáles cree que son los desafíos para articular mejor las acciones de las comunidades Indígenas y el sector salud, para proteger la salud de las personas en momentos de un evento climático extremo?
14. ¿Cómo cree que se podría articular mejor las acciones de las comunidades y lideres indígenas con el personal de salud o el centro de salud para enfrentar mejor los peligros hacia la salud en los momentos de un evento climático extremo?
    - 14.1. Puede brindarnos al menos dos sugerencias para mejorar la articulación (si no responde, se le puede mencionar: en términos de información, coordinación, liderazgo, financiamiento)
15. ¿Con otros sectores, que no sean salud, (educación, ambiente, cultura), cómo cree que se podría articular mejor las acciones de las comunidades y lideres indígenas para enfrentar mejor los peligros hacia la salud en los momentos de un evento climático extremo?
    - 15.1. Puede brindarnos al menos dos sugerencias para mejorar la articulación (si no responde, se le puede mencionar: en términos de información, coordinación, liderazgo, financiamiento)
