# Supplementary material for: Pathways to strengthen the climate resilience of health systems in the Peruvian Amazon by working with Indigenous leaders, communities and health officers
Source: BMJ Glob Health. 2024 Sep 7;8(Suppl 3):e014391. doi: 10.1136/bmjgh-2023-014391 (PMC11733073; doi:10.1136/bmjgh-2023-014391)
Supplement: online supplemental file 2 [file bmjgh-8-Suppl_3-s002.pdf]

1. List of codes created a priori

Codes	Description
Current climatic shocks	Climatic events relevant to health systems in each Amazon region
Exposure	The presence of social and natural systems in the location of interest that are affected by climatic events. It includes people, livelihoods, ecosystems, cultural resources, and infrastructure, among others and responds to the questions
Responses	The responses of the official and Indigenous health systems facing climatic shocks to protect the health of their patients, health workforce, health care facilities, general population or other relevant economic, social, or cultural resources in places susceptible to be negatively affected by climate-related hazards.
Anticipate	The capacity or ability of the health system (Indigenous or official) to identify a potential climatic shock (before the shock)
Respond	The capacity or ability of a health system to protect health in the face of climatic shocks (during the shock)
Cope	The capacity or ability of a health system to continue to deliver the same level (quantity, quality and equity) of basic healthcare services and protection to populations despite the shock using the same level of resources and capacities (during the shock)
Recover	The capacity or ability of a health system to recover its functions in the mid-term (after the risk)
Adapt	The capacity or ability of a health system to recover its functions and take advantage of the situation in the long term (after the risk)
Transform	The capacity or ability of a health system to transform its structure, some functions, and values as a result of the climatic shock, but by keeping their main function in this case delivering health care. Transformation implies a learning process , self-organisation, and change
Challenges	Difficulties to manage and respond to climatic shocks
Vulnerabilities	The propensity of the system to be damaged by the shock
Health Risks	Direct or indirect result of the hazard or shock on the health of the population
Amplifiers	Social, economic, political, environmental, and cultural conditions that increase the health risks posed by climate change, and thus increase the state of vulnerability
Modulators	Social, economic, political, environmental, and cultural conditions that reduce the health risks posed by climate change, and thus change the state of vulnerability

Building Blocks	Six core components or building blocks of health systems
Essential medical products and technologies	This component refers to adapting health infrastructure, technologies, and processes to climate risks, promoting new technologies to increase climate resilience, and improving the sustainability of health operations
Financing	This component aims to establish health-specific funding mechanisms, develop funding in sectors influencing health, and increase access to climate change funding streams at a national level.
Health information systems	This building block includes three sub-components: 1) vulnerability, capacity, and adaptation assessments, 2) integrated risk monitoring and early warning, and 3) health and climate research.
Health workforce	This building block refers to strengthening the technical, professional, and organisational capacity of health personnel and health systems for climate change and health management. This component aims to implement measures for 1) human resource skill building, training, and education, 2) organisational capacity development, and 3) communications and awareness raising.
Leadership and governance	This building block refers to incorporating climate change-related impacts into health policy or actions within the formal health system and in health-determining sectors.
Service delivery	This building block includes three sub-components: 1) emergency preparedness and management, 2) management of environmental determinants of health, and 3) climate-informed health programs.

2. List of codes created after revising the interviews

**Amplifiers**	**Social, economic, political, environmental, and cultural conditions that increase the health risks posed by climate change, and thus increase the state of vulnerability**
Agriculture situation	The agricultural situation amplifies the consequences of climate hazards
Deforestation	Deforestation in Indigenous communities increases the risk of climate hazards
Intensive agriculture	Monoculture and intensive agriculture
Loss of biodiversity	Loss of biodiversity in Indigenous communities, which may worsen the consequences of climate hazards
Loss of cultural values	The loss of Indigenous cultural values could make them less resilient to climate change
Loss of water resources	Loss of water resources in Indigenous communities, which may worsen the consequences of climate hazards
Oil companies	Oil activities in Indigenous territories or affecting Indigenous peoples
Pollution	Pollution in Indigenous communities and their surroundings may worsen the consequences of climate hazards
**Challenges of the responses**	**Difficulties in managing climatic shocks**
Cultural diversity & different livelihoods	Indigenous peoples have different livelihoods, health, and food systems than in urban areas
Difficult coordination with Indigenous communities	It is difficult for the official health system officers to coordinate with Indigenous communities during climate emergencies
Financing	Insufficient financing for the responses to climate change hazards
Insufficient health workers	Insufficient health workers to assist Indigenous communities

Insufficient or late support	The responses of the official health systems were perceived as insufficient or late
Lack of anticipation	There are insufficient anticipation measures to prevent health risks facing climate hazards
Lack of intercultural and territorial perspective	Official health system lacks responses with an intercultural and territorial approach, adequate to different cultural settings and geographies, respectively
Unimportance of climate change	Official health systems dedicate little importance to climate change
Unwilling to leave places of risk	The population is not willing to leave places of risk
**Current Climatic Shocks or Hazards**	**Climatic events relevant to health systems in each Amazon region**
Changes in seasonality	Changes in seasonality
Cold waves	Cold waves
Droughts	Droughts
Earthquake	Earthquake
Exceptional precipitations	Exceptional precipitations
Floodings	Floodings
Forest fire	Forest fire
Heatwaves or higher temperature	Heatwaves or higher temperature
Landslides	Landslides
No changes in seasonality	The interviewees do not perceive any change in seasonality
Rain and sun	Abrupt change of the weather, combining rain and sun

Time since the climate variability	Time since the climate variability
Winds	Winds
**Exposure**	**The presence of social and natural systems in the location of interest that are affected by climatic events. It includes people, livelihoods, ecosystems, cultural resources, and infrastructure, among others, and respond to the questions**
Biodiversity	Biodiversity, namely, plants, wild animals and fruits
Children	Children
Crops	Crops
Education infrastructure	Education infrastructure
Elderly	Elderly
Farm animals	Farm animals
Farmers	Farmers
Health facilities	Health facilities
Houses	Houses
Indigenous communities	Indigenous communities
River communities	River communities
Rural communities	Rural communities
Transportation	Transportation

**Health**	**About Indigenous and Official Health Systems**
COVID-19	About COVID-19 pandemic
Frequent diseases	Most frequent diseases in Indigenous communities
Indigenous health system	Indigenous health system
Indigenous community health workers	About the Indigenous community health workers
Indigenous healthcare and medicine	Indigenous healthcare and medicine
Non-western diseases	Diseases that are not recognised by the official health system, for example, *mal de ojo* or *brujería*
Official health system	Official health system
Approach to Indigenous health systems	The linkages official health system does with Indigenous health systems
Desired qualities for health workers	Desired qualities for health workers
Inadequate assistance in official healthcare	Inadequate assistance in official healthcare
Lack of trust in official healthcare	Indigenous peoples do not trust official healthcare
Official healthcare	
Recommendations for official healthcare	Recommendations for official healthcare

Trust in official healthcare	Indigenous peoples trust in official healthcare
Prioritized diseases	What are the diseases that should be prioritized in Indigenous communities?
What is health	What is health for Indigenous participants?
**Health Risks and Related Impacts**	**Result of the hazard or shock and how the health system is responding to the hazard, to protect the health of their patients, health workforce, health care facilities, general population, or other relevant economic, social, or cultural resources in places susceptible to be negatively affected by climate-related hazards.**
Airborne diseases	Airborne diseases
Deaths	Deaths
Direct injures	Direct injures
Food insecurity	Food insecurity
Headache or fever	Headache or fever
Infections	Infections
Mental health	Mental health
Skin diseases	Skin diseases
Snake bites	Snake bites
Vector-borne diseases	Vector-borne diseases
Waterborne diseases	Waterborne diseases
Water insecurity	Water insecurity

**Knowledge about climate change**	**What do the participants know and understand about climate change?**
**Modulators**	**Social, economic, political, environmental, and cultural conditions that reduce the health risks posed by climate change, and thus change the state of vulnerability**
Forest	Forest protects the community from severe consequences of climate hazards
**Recommendations for the responses**	**Recommendations for the responses**
Better articulation among actors	Better articulation between sectors (health, environment, local government, etc.), and also with private actors (NGOs)
Better articulation with Indigenous representatives	Better articulation and communication with Indigenous representatives before, during and after climate hazards
More financing, resources and infrastructure	More financing, resources and infrastructure to prevent and assist during climate hazards
More training on climate change	More training on climate change
Preventive and early responses	Implement preventive and early responses, to climate hazards
Promote Indigenous medicine	Promote Indigenous medicine to protect health during climate hazards
Strengthen leadership	Strengthen leadership to prevent and assist climate hazards
**Responses**	**The responses of the official and Indigenous health systems facing climatic shocks**
Adapt	The capacity or ability of a health system to recover its functions and take advantage of the situation in the long term (after the risk)
Adapt health facilities and infrastructure	Adapt health facilities and infrastructure to be better prepared to face climate hazards

Agriculture adaptation	Indigenous health systems adapt their agriculture practices to the climate conditions
Climate change governance	Climate Change in national or regional governance
Daily healthy practices	Indigenous people incorporate healthy practices in daily life to maintain health and be more vital if a climate hazard occurs
Environmental Health	Official health systems have a working group or office for environmental health
Fish farms	Implement farms in Indigenous communities to eradicate food insecurity and support the economy
Malaria community plan	Malaria community plan of official health system
Protect the territory and the forest	Protect the territory and the forest with the aim to reduce the consequences of climate hazards
Reforestation in the communities	Reforestation to reduce the consequences of climate hazards
Risk management & climate change	Risk management adapts to the climate change scenario
Spatial organization of the territory	Spatial organization of the territory to protect the forest and prevent negative consequences of climate hazards
Stay away from the river	The Indigenous community stays away from the river to avoid being affected by floodings
Training in healthy practices	Official health system trains Indigenous peoples to incorporate healthy practices to prevent health impacts during possible future climate hazards
Training in prevention	Official health system trains Indigenous people on how to prevent climate hazards having severe consequences
Anticipate	The capacity or ability of the health system (Indigenous or official) to identify a potential climatic shock (before the shock)
River defense	Create river defense to avoid the consequences of flooding
Ensure medicine provision	The official health system ensures the provision of medicine to prevent climate hazard from occurring

Indigenous medicine	Indigenous people use their medicine to cope with health risks caused by climate hazards
Information about climate hazards	Inform about possible climate hazards
Prediction of climatic events	Indigenous communities know how to prevent possible climate hazards
Risk management	The anticipation measures the local risk management offices take
Waste management & cleaning	Adequately manage waste and clean the community to prevent negative consequences of climate hazards
Coordination between sectors and other actors	How is the coordination between sectors and other actors regarding climate hazards?
Coordination with Indigenous communities	How is the coordination between official and Indigenous health systems regarding climate hazards?
Cope	The capacity or ability of a health system to continue to deliver the same level (quantity, quality, and equity) of essential healthcare services and protection to populations despite the shock using the same level of resources and capacities (during the shock)
Food from the forest or river	Indigenous peoples rely on food from the forest or rivers when they lose their crops due to climate hazards
Healthy practices	Healthy practices Indigenous peoples take to avoid health risks during climate hazards, for example, keeping themselves warm during cold waves
Indigenous medicine	Use Indigenous medicine to cope with health risks provoked by climate hazards
Maintain health assistance	Official health systems maintain health assistance during climate hazards
Store food	Indigenous peoples store food
No response from the official health system	The official health does not provide any response to tackle climate hazards
Recover	The capacity or ability of a health system to recover its functions in the mid-term (after the risk)

Cash aid	Cash aid for those affected by climate hazards
Clean the landslide or flooding	The Indigenous or official health system cleans the community after the climate hazard
Food aid	Food aid for those affected by climate hazards
Provide materials or infrastructure	Provide materials or infrastructure for those affected by climate hazards
Rebuild their houses	Indigenous communities rebuild their houses affected by the climate hazards
Relocate the community	Relocate the community that is in a risk place
Share or exchange food and seeds	Indigenous people share or exchange food and seeds after being affected by climate hazards
Time to recover	How long does it take to recover from the climate hazard?
Respond	The capacity or ability of a health system to protect health in the face of climatic shocks (during the shock)
chlorinate and monitor water	chlorinate and monitor water quality for those affected by climate change
Evacuated risk area	Indigenous peoples by themselves or with the support of the official health system evacuate risk areas affected by climate hazards
Give warm cloth	The official health system provides warm clothes, so Indigenous peoples are protected from the cold
Plataforma Defensa Civil	The responses of the ‘Defensa Civil’ platform and the multisectoral space for emergencies
Provide additional medicine and health assistance	The official health system provides additional medicine, medical implements, and health assistance to respond to the population's needs during climate hazards.
Provide water and food	Provide water and food for those affected by climate hazards
Register the event and the affected	Register the event and the affected

Transform	The capacity or ability of a health system to transform its structure, functions, and values because of the climatic shock
**Vulnerabilities**	**The propensity of the system to be damaged by the climate shock**
Food insecurity	Previous food insecurity situation among Indigenous peoples could worsen with climate hazards
Inadequate public infrastructure	Inadequate public infrastructure, including health and transportation
Lack of public water and sewage	Lack of public water and sewage
Land insecurity	Insecurity of Indigenous peoples' ownership of their territories
Other health risks	Previous and current health risks that Indigenous peoples face and that could be worsened by climate hazards
Poor connectivity	Poor connectivity
Poverty	Poverty
Remoteness	The remoteness and difficulty access to Indigenous communities
