# Supplementary material for: Pathways to strengthen the climate resilience of health systems in the Peruvian Amazon by working with Indigenous leaders, communities and health officers
Source: BMJ Glob Health. 2024 Sep 7;8(Suppl 3):e014391. doi: 10.1136/bmjgh-2023-014391 (PMC11733073; doi:10.1136/bmjgh-2023-014391)
Supplement: online supplemental file 3 [file bmjgh-8-Suppl_3-s003.pdf]

Supplementary Material 3

Questionnaire for the health facility (Spanish)

Cuestionario para el establecimiento de salud

Objetivo: Caracterizar el sistema de salud, su capacidad para responder a eventos climáticos (o peligros), y cómo searticula con la respuesta de las poblaciones Indígenas

Participantes: Se entrevistará al responsable del establecimiento de salud o a alguien que se designe, y que conozca lainformación sobre el centro de salud.

Tiempo estimado del cuestionario: 1 hora

Antes de iniciar se debe corroborar que el participante este cómodo, y haya brindado su consentimiento para sergrabado o para tomar notas. Las peguntas se aplicarán en español. Se grabará sólo si el participante lo autoriza.

A. Infraestructura y tecnología que cuenta el servicio de salud

1. Código del establecimiento de salud________
2. Nivel de atención del establecimiento de salud _____________
3. Altura y punto de GPS________________________(lo tenemos registrado)
4. Ubicación del establecimiento de salud en relación al centro urbano más cercano:
   - Muy remoto
   - Remoto
   - Cercano
5. ¿El establecimiento de salud cuenta con energía eléctrica?
   - Si, las 24 horas
   - Si, sólo por_horas al día (aprox)
   - No

   Marcar cuál es la fuente de energía eléctrica que usan en el establecimiento de salud

   5.1. Energía solar
   5.2. Energía de generador eléctrico que funciona con gasolina o petróleo
   5.3. Energía de la red pública
   5.4. Otro _____________
6. ¿El establecimiento de salud cuenta con agua para **lavarse** las manos?
   - Si, las 24 horas
   - Si, sólo por____horas al día (aprox)
   - No

   Marcar cuál es la fuente del agua que usan para **lavarse** las manos en el establecimiento de salud

   6..1. Agua de servicio público
   6..2. Agua entubada
   6..3. Agua de lluvia
   6..4. Agua de pozo
   6..4. Agua de Rio
   6..5. Agua de quebrada
   6..6. Agua de botella de plástico o agua tratada

6..7. Otro ____

7. ¿El establecimiento de salud cuenta con agua potable para **tomar**?
   - Si, las 24 horas
   - Si, sólo por____horas al día (aprox)
   - No

   Anotar cuál es la fuente del agua que usan mayormente para tomar en el establecimiento de salud

   7..1. Agua de servicio público
   7..2. Agu entubada
   7..3. Agua de lluvia
   7..4. Agua de pozo
   7..4. Agua de Rio
   7..5. Agua de quebrada
   7..6. Agua de botella de plástico o agua tratada
   7..7. Otro ____

8. ¿El establecimiento de salud cuenta con servicios higiénicos **para hacer sus necesidades** que funcionan actualmente?
   - Si, las 24 horas
   - Si, sólo por_horas al día (aprox)
   - Si, pero no funcionan actualmente
   - No

   Marcar cuál es el tipo de servicio que usan para hacer sus necesidades en el establecimiento de salud

   8..1. Servicio publico
   8..2. Letrina
   8..3. A campo abierto
   8..4. Rio
   8..5. Otro

9. ¿El establecimiento de salud cuenta con servicios higiénicos **para bañarse** y asearse que funcionanactualmente?
   - Si, las 24 horas
   - Si, sólo por____horas al día (aprox)
   - Si, pero no funcionan actualmente
   - No

   Marcar cuál es el tipo de servicio que usan para **bañarse** en el establecimiento de salud

   9..1. Servicio publico
   9..2. Letrina
   9..3. A campo abierto 9..4. Rio
   9..5. Otro

10. ¿El establecimiento de salud cuenta con un sistema de **comunicación** para emergencias?
    - Si, las 24 horas
    - Si, sólo por____horas al día (aprox)
    - No

    Marcar cuál es el tipo de servicio que usan mayormente para la

comunicación en emergencias

10..1. Celular
10..2. radiofonía
10..3. Otro

11. ¿El establecimiento de salud cuenta con **internet** para el uso del personal de salud?
    - Si, las 24 horas
    - Si, sólo por___horas al día (aprox)
    - No

    Marcar como aceden al servicio de internet

    11..1. Propio del centro de salud
    11..2. Ofrecido por la municipalidad
    11..3. Pagado por cada personal de salud
    11..4. Otro

12. ¿El establecimiento de salud cuenta con **un sistema de manejo de desechos comunes (basura nocontaminada como botellas, bolsas plásticas, papeles, etc)**?
    - Si, las 24 horas
    - Si, sólo por___horas al día (aprox)
    - No

    Marcar como manejan los desechos comunes

    12..1. Lo recoge el servicio municipal
    12..2. Lo recoge otro servicio publico
    12..3. Lo recoge un servicio pagado por el establecimiento de salud
    12..4. Lo queman
    12..5. Lo entierran
    12..6. Otro _________________

13. ¿El establecimiento de salud cuenta con **un sistema de manejo de desechos biológicos (basuracontaminada que contiene sangre, gasa, u otros fluidos como secreciones de limpiar heridas, esputo, orina, y equipos como agujas, jeringas y otros materiales que se usa en el trabajo de atender a un paciente)?.**
    - Si, las 24 horas
    - Si, sólo por___horas al día (aprox)
    - No

    Marcar como manejan los desechos comunes

    13..1. Lo recoge el servicio municipal
    13..2. Lo recoge otro servicio publico
    13..3. Lo recoge un servicio pagado por el establecimiento de salud
    13..4. Lo queman
    13..5. Lo entierran
    13..6. Otro

14. ¿El establecimiento está diseñado de acuerdo a las normas técnicas de infraestructura?
    - Si

- No
- No sabe

15. ¿Usted aplica o conoce el manejo de la bioseguridad en salud? Por ejemplo, para para separar los desechos sanitarios
    - Si
    - No

B. De la prestación del servicio

16. ¿Cuántas comunidades atiende este centro de salud? _____
17. De este total, ¿cuántas son comunidades Indígenas?
18. ¿Cuántas personas aproximadamente están en la jurisdicción del establecimiento de salud? ___________________________
19. ¿Cuántas personas diría usted aproximadamente son adicionales como visitantes, flotantes, o migrantes que se atienden en este establecimiento de salud (e.j. el año pasado 2022)? _
    - Cero al año
    - Entre 1 a 20 al año
    - Entre 21 a 50 al año
    - Mas de 50 al año
20. ¿Qué tipo de profesionales y técnicos laboran (o apoyan el trabajo) en este establecimiento de salud?
    - médicos__________(anotar cuantos)
    - enfermeros (as)__________(anotar cuantos)
    - obstetras__________(anotar cuantos)
    - nutricionistas__________(anotar cuantos)
    - dentistas u odontólogos (as)__________(anotar cuantos)
    - técnico (ca) de enfermería__________(anotar cuantos)
    - técnico (ca) de laboratorio__________(anotar cuantos)
    - Cocinero (a)__________(anotar cuantos)
    - Vigilante (a)__________(anotar cuantos)
    - Biólogo (a)__________(anotar cuantos)
    - Administrador (a)__________(anotar cuantos)
    - Otro _________

21. ¿Qué enfermedades o males diría usted que son los más frecuentes en su establecimiento de salud?

Mencionar al menos tres:

- ____
- ____
- ____

22. De estos que mencionó ¿Cuál diría usted que es el más importante y debe ser atendido como prioridad?

- ____

Por favor explicar porque le parece importante

C. De las atenciones brindadas

Las siguientes preguntas son en relación con la atención de las enfermedades nutricionales, y las actividades que realizan para garantizar la seguridad alimentaria y nutricional de la comunidad en su zona. La seguridad alimentaria y nutricional se define como el estado "*en el cual todas las personas gozan, en forma oportuna y permanente, de acceso físico, económico y social a los alimentos que necesitan, en cantidad y calidad, para su adecuado consumo y utilización biológica, garantizándoles un estado de bienestar general que coadyuve allogro de su desarrollo"*

23. ¿A su parecer existe suficiente personal para *atender la seguridad alimentaria y nutricional* en la zonadonde usted trabaja?
    - Si
    - No,
24. ¿A su parecer el personal que atiende está suficientemente capacitado para para *atender la seguridadalimentaria y nutricional* en la zona donde usted trabaja?
    - Si
    - No,
25. ¿A su parecer el sector salud tiene suficiente liderazgo para *atender la seguridad alimentaria y nutricional*

en la zona donde usted trabaja?
    - Si
    - No,
26. ¿A su parecer existe la normativa clara para *atender la seguridad alimentaria y nutricional* en su región?
    - Si
    - No,
27. ¿A su parecer el personal de en su zona, tiene suficiente:
    - Tecnología incluye: balanza, tallímetro, hemoglobinómetro para atender la situación nutricional de la población en su jurisdicción para atender esta condición Si () No () No sabe ()
    - Vacunas para prevenir enfermedades de la infancia: Si () No () No sabe ()
    - Medicinas Suplementos de hierro Si () No () No sabe ()
    - Medicinas: Antiparasitarios Si ( ) No ( ) No sabe ( )
    - Pruebas diagnósticas para descarte de anemia Si ( ) No ( ) No sabe ( )
    - Pruebas diagnósticas para descarte de malaria Si ( ) No ( ) No sabe ( )
    - Pruebas diagnósticas para descarte de dengue Si ( ) No ( ) No sabe ( )

D. Sobre la llegada de del sector salud con la población indígena, para atender la seguridad alimentaria y nutricional en su jurisdicción

28. ¿Qué tan buena es la comunicación con la población indígena:
    - Muy Buena
    - Buena
    - Regular
    - Mala
    - Muy Mala
29. Qué tanta aceptación tiene las intervenciones del sector salud en la población

indígena?

- Muy Buena
- Buena
- Regular
- Mala
- Muy Mala

30. ¿A su parecer el sector salud en su zona, tiene suficiente financiamiento para *atender la seguridadalimentaria y nutricional* en su región?
    - Si
    - No
31. ¿A su parecer sector salud necesitaría apoyo de otros sectores para *atender la seguridad alimentaria ynutricional* en su región en su zona? ¿Cuáles sectores serian de importancia de involucrar y por qué?

- ____________________
- ____________________
- ____________________
- ____________________

De las coordinaciones con la comunidad, **,** los agentes comunitarios de salud, y los agentes de medicina tradicional

32. ¿El establecimiento de salud coordina con agentes **comunitarios** de salud (o promotores de salud)?
    - Si, constantemente
    - Si, a veces
    - Si, sólo para ciertas actividades
    - No
33. Si, la respuesta es sí, ¿de qué forma se da la coordinación? (leer las siguientes y marcar todo lo quecorresponde)
    - Si , para monitorear el caso de emergencia
    - Si , para seguimiento de gestantes
    - Si , para seguimiento de condiciones nutricionales
    - Si , para seguimiento de malaria
    - Si, para brindar información de prevención
    - Si, para vacunar
    - Si, para entregar tratamientos
34. Si, la respuesta es sí, ¿ por qué medios se da la comunicación?
    - Si , por telefono
    - Si , por radiofonía
    - Si , en persona
    - Si , otro anotar
35. Si, la respuesta es sí ¿El establecimiento de salud brinda capacitación a **sus agentes comunitarios de salud**?
    - Si
    - No

Si, la respuesta es si

36. ¿Cuántas veces año aproximadamente (en el año 2022)?
    - 1 vez al año
    - 2 veces al año

- Mas de 2 veces al año

37. ¿El establecimiento de salud coordina con agentes de **medicina tradicional** como curanderos oparteras)?
    - Si, constantemente
    - Si, a veces
    - Si, sólo para ciertas actividades: anotar las actividades _ yanotar el tipo de agentes de medicina tradicional con los que coordina
    - No___(¿por qué?)
38. Si, la respuesta es sí, marcar con que tipo de agentes de medicina tradicional coordina:
    - Partera
    - Partero
    - Curandera
    - Curandero
    - Otro_________

39. Si, la respuesta es sí, ¿de qué forma se da la coordinación? (leer las siguientes y marcar todo lo quecorresponde)
    - Si , para monitorear el caso de emergencia
    - Si , para seguimiento de gestantes
    - Si , para seguimiento de condiciones nutricionales
    - Si , para seguimiento de malaria
    - Si, para brindar información de prevención
    - Si, para vacunar
    - Si, para entregar tratamientos

40. Si, la respuesta es sí, ¿por qué medios se da la comunicación?
    - Si , por teléfono
    - Si , por radiofonía
    - Si , en persona
    - Si , otro anotar_______________
41. Si, la respuesta es sí ¿El establecimiento de salud brinda capacitación a **sus agentes de medicina tradicional**?
    - Si
    - No

Si, la respuesta es si

42. ¿Cuántas veces año aproximadamente (en el año 2022)?
    - 1 vez al año
    - 2 veces al año
    - Mas de 2 veces al año
43. ¿El establecimiento de salud coordina con el jefe de la comunidad y su comitiva de salud?
    - Si
    - no
44. ¿En su establecimiento de salud hay al menos un personal capacitado en salud intercultural?
    - Si
    - No

Si, la respuesta es si, ¿qué tipo capacitación ha recibido?

____

45. ¿En su establecimiento de salud hay personal que habla el idioma indígena?
    - Si
    - No

Si, la respuesta es si, ¿qué nivel de fluidez tiene para comunicarse ?

- básico
- fluido

Despedirse, darles gracias, asegurarle que la información será tratada con la mayor confidencialidad.

Entregar la hoja informativa sobre seguridad alimentaria y nutricional.

Preguntar si desea ser contactado para invitarle el curso corto sobre cambio climático y salud, que organizaremos entre marzo y julio 2023. Sí responde que si, entonces pedir algún contacto para avisarle. Anotar y guardar la información de manera segura y sin riesgo de que se pierda

---
