# Supplementary material for: Pathways to strengthen the climate resilience of health systems in the Peruvian Amazon by working with Indigenous leaders, communities and health officers
Source: BMJ Glob Health. 2024 Sep 7;8(Suppl 3):e014391. doi: 10.1136/bmjgh-2023-014391 (PMC11733073; doi:10.1136/bmjgh-2023-014391)
Supplement: online supplemental file 4 [file bmjgh-8-Suppl_3-s004.pdf]

Participants in the Loreto workshop of dissemination of the research results

#	Health system	Occupation	Sex
1	Official health system	Nurse technician in a health post	Man
2	Indigenous health system	Indigenous representative	Man
3	Indigenous health system	Indigenous representative	Man
4	Indigenous health system	Indigenous representative	Man
5	Official health system	Nurse in a health post	Woman
6	Official health system	Obstetrician	Woman
7	Official health system	Nurse	Woman
8	Official health system	Nurse technician	Man
9	Indigenous health system	Indigenous representative	Man
10	Official health system	Obstetrician	Woman

Participants in the Junin workshop of dissemination of the research results

#	Health system	Occupation	Sex
1	Indigenous health system	Community health agent	Woman
2	Indigenous health system	Community health agent	Man
3	Indigenous health system	Community health agent	Man
4	Indigenous health system	Community health agent	Woman
5	Official health system	Environmental Office in the health network	Man
6	Official health system	Indigenous Office in the Health Network	Man
7	Indigenous health system	Community health agent	Woman
8	Official health system	Obstetrician in a health post	Woman
9	Official health system	Mental health office in the health network	Man
10	Official health system	Laboratory technician	Man
11	Official health system	Nurse technician in a health post	Woman
12	Official health system	Local government	Man

13	Indigenous health system	Community health agent	Woman
14	Indigenous health system	Community health agent	Woman
15	Official health system	Obstetrician in a health post	Woman
16	Official health system	Obstetrician in a health post	Woman
17	Indigenous health system	Indigenous representative	Woman
18	Indigenous health system	Community health agent	Woman
19	Indigenous health system	Indigenous representative	Woman
20	Official health system	Obstetrician in a health post	Woman
21	Indigenous health system	Indigenous representative	Woman
22	Official health system	Nurse technician in a health post	Man
