# Supplementary material for: Pathways to strengthen the climate resilience of health systems in the Peruvian Amazon by working with Indigenous leaders, communities and health officers
Source: BMJ Glob Health. 2024 Sep 7;8(Suppl 3):e014391. doi: 10.1136/bmjgh-2023-014391 (PMC11733073; doi:10.1136/bmjgh-2023-014391)
Supplement: online supplemental file 5 [file bmjgh-8-Suppl_3-s005.pdf]

Table 1. Responses of the official and Indigenous health systems to climate change in Loreto and Junin regions according to the WHO building blocks

Health system building block	Responses	
	**Official health system**	**Indigenous health system**
**Leadership and governance**	● Policy roadmaps for implementing health adaptation measures in Loreto and Junin by 2025<br>● Regional Strategies for Climate Change developed by Loreto and Junin regional governments (pending to be updated)<br>● Disaster Risk Management offices established within health networks<br>● The Junin and Loreto regional strategy for climate change proposes coordination with public and private actors.	● Participation of Indigenous organizations in a climate change governance platform at the national level<br>● Participation of Indigenous organizations and community health agents in the implementation of health interventions led by health networks
**Emergency preparedness and management**	● Intersectoral platforms provide water, food, warm clothes, and health assistance during climate events.<br>● Emergency brigades led by health networks provide first aid, vaccination, and relocate affected communities<br>● Contingency plans are implemented by disaster risk management offices in health networks	● Building river defenses in zones prone to inundations<br>● Mechanisms to evacuate risky zones based on previous experiences from climate disasters<br>● Using Indigenous medicine to prevent and manage climate-sensitive health risks<br>● Adopting daily healthy behaviors to protect communities from climate hazards (e.g. keeping warm during cold waves)<br>● Organizing their communities to clean and restore houses and other affected infrastructures after climate events

**Management of environmental determinants of health**	• Environmental health offices in regional health networks monitor water and air quality and other environmental factors influencing health<br>• Peru's NAP has established an adaptation measure in the health sector aimed to transfer healthy practices to vulnerable families toward vector-borne, water-borne, and food-borne diseases, and extreme temperatures.	• Protecting the forests through reforestation interventions along with the official health system (public and private organizations)<br>• Performing waste management and cleaning interventions in communities to prevent climate-sensitive diseases (e.g. vector-borne diseases)
**Climate-informed health programs**	• Health networks train communities on healthy practices to prevent climate-sensitive diseases<br>• Health networks develop health programmes targeted to climate-sensitive diseases (e.g. malaria)	• Adopting daily healthy behaviors to protect communities from climate hazards (e.g. keeping warm during cold waves)<br>• Modifying agriculture and livestock practices and healthcare and households infrastructure according to climate conditions
**Vulnerability and adaptation assessment**	• The assessment of the exposure, vulnerability and climate hazards allowed to define a level of risk for each province and district in Loreto and Junin regions.<br>• To date, no adaptation assessment has been performed yet at a national or regional level.	• N/A
**Integrated risk monitoring and early warning**	• Health networks share information on imminent climate hazards with health posts and communities to anticipate health assistance	• Using Indigenous knowledge to predict climate events<br>• Providing information to the official health system about affected communities and local health needs during climate hazards

**Health and climate research**	• At the regional level, the Junin and Loreto regional strategies for climate change highlight the need for strengthening research capacity to enable the implementation of adaptation measures in each region	• Indigenous knowledge about medicine and climate hazards is build up on transmitting wisdom through generations and learning from past experiences.
**Climate resilient and sustainable technologies and infrastructure**	• Local governments provide food, materials, and cash aid to affected communities<br>• In both Loreto and Junin, the need for research on new technologies has been identified as an enabling condition for the implementation of adaptation strategies in vulnerable healthcare services<br>• Loreto regional strategy for climate change, indicates that financial mechanisms are required to identify and prioritize healthcare establishments for the diagnosis and implementation of clean technologies.	• Exchanging and sharing foods and seeds to protect food security after climate events<br>• Modifying agriculture and livestock practices and healthcare and households infrastructure according to climate conditions<br>• Relying on foods from the forests and rivers after climate events to protect food security<br>• Participating in new technologies to adapt food systems to climate change (e.g. fish farms, garden projects)
**Health workforce**	• Disseminating information on climate change to improve adaptive capacities to climate change.<br>• Training of the health workforce on research about climate change and health	• N/A
