# Supplementary material for: Pathways to strengthen the climate resilience of health systems in the Peruvian Amazon by working with Indigenous leaders, communities and health officers
Source: BMJ Glob Health. 2024 Sep 7;8(Suppl 3):e014391. doi: 10.1136/bmjgh-2023-014391 (PMC11733073; doi:10.1136/bmjgh-2023-014391)
Supplement: Abstract translation 1 [file bmjgh-8-Suppl_3-s006.docx]

**Antecedente:** El conocimiento y las respuestas Indígenas fueron implementados durante la pandemia de COVID-19 para proteger la salud, mostrando como la participación de las comunidades Indígenas en los sistemas de salud podría ser una vía para aumentar la resiliencia de los sistemas de salud a los peligros emergentes tales como los impactos del cambio. Este estudio tuvo como objetivo informar la resiliencia de los sistemas de salud mediante i) examinar si y cómo el sistema de salud en la Amazonía peruana se está adaptando al cambio climático y ii) comprender cómo las respuestas de las comunidades y líderes Indígenas a los peligros climáticos se articulan con el sistema de salud oficial.

**Métodos:**

Este estudio se realizó en dos redes de salud de la Amazonía peruana que brindan servicios a comunidades Indígenas en las regiones de Junín y Loreto. Se realizó un diseño convergente de metodología mixta utilizando una encuesta (13 establecimientos de salud), entrevistas semiestructuradas (27 participantes de los sistemas de salud oficiales y 17 participantes del sistema de salud Indígena), y dos talleres presenciales para validar y seleccionar prioridades para la mejora de los sistemas de salud (32 participantes). Utilizamos un marco teórico de sistemas de salud resilientes al clima, y los componentes básicos de los sistemas de salud de la OMS.

**Resultados:** Los sistemas de salud Indígena y oficial en la Amazonía peruana se están adaptando al cambio climático. Las respuestas Indígenas incluyen el conocimiento Indígena sobre la variabilidad del clima, el uso de medicina “vegetal” para gestionar los riesgos para la salud y redes sociales para compartir alimentos y otros recursos. Las respuestas del sistema de salud oficial incluyen estrategias y plataformas de respuesta que actúan principalmente después de la ocurrencia de peligros climáticos. Las principales vías para articular los sistemas de salud Indígena y oficial incluyen la incorporación de representantes Indígenas en la gobernanza del clima y la salud, la capacitación de los trabajadores de salud, la mejora de la prestación y el acceso a servicios, el fortalecimiento de la evidencia para respaldar las respuestas Indígenas y el aumento del presupuesto para las respuestas a emergencias climáticas.

**Conclusiones:** Las principales vías de resiliencia exigen un cambio de paradigma en los sistemas de salud que reconozca el valor de la resiliencia Indígena para la adaptación en salud, promueva un enfoque más participativo en el sistema de salud y amplíe la percepción de la salud como una dimensión intrínsecamente ligada al medio ambiente.
